# *Arabidopsis thaliana FLA4* functions as a glycan‐stabilized soluble factor via its carboxy‐proximal Fasciclin 1 domain

**DOI:** 10.1111/tpj.13591

**Published:** 2017-06-13

**Authors:** Hui Xue, Christiane Veit, Lindy Abas, Theodora Tryfona, Daniel Maresch, Martiniano M. Ricardi, José Manuel Estevez, Richard Strasser, Georg J. Seifert

**Affiliations:** ^1^ Department of Applied Genetics and Cell Biology University of Natural Resources and Life Science BOKU Vienna Muthgasse 11 A‐1190 Vienna Austria; ^2^ Department of Biochemistry University of Cambridge Cambridge CB2 1QW UK; ^3^ Department of Chemistry University of Natural Resources and Life Science BOKU Vienna Muthgasse 11 A‐1190 Vienna Austria; ^4^ Biología Molecular y Neurociencias–Consejo Nacional de Investigaciones Científicas y Técnicas(IFIByNE‐CONICET) Instituto de Fisiología Facultad de Ciencias Exactas y Naturales Universidad de Buenos Aires Buenos Aires C1428EGA Argentina; ^5^ Fundación Instituto Leloir and Instituto de Investigaciones Bioquímicas de Buenos Aires Buenos Aires CP C1405BWE Argentina

**Keywords:** arabinogalactan protein, fasciclin, GPI‐anchor, *N‐*glycan, *O‐*glycan, *Arabidopsis thaliana*

## Abstract

Fasciclin‐like arabinogalactan proteins (FLAs) are involved in numerous important functions in plants but the relevance of their complex structure to physiological function and cellular fate is unresolved. Using a fully functional fluorescent version of *Arabidopsis thaliana* FLA4 we show that this protein is localized at the plasma membrane as well as in endosomes and soluble in the apoplast. FLA4 is likely to be GPI‐anchored, is highly *N‐*glycosylated and carries two *O‐*glycan epitopes previously associated with arabinogalactan proteins. The activity of FLA4 was resistant against deletion of the amino‐proximal fasciclin 1 domain and was unaffected by removal of the GPI‐modification signal, a highly conserved *N‐*glycan or the deletion of predicted *O‐*glycosylation sites. Nonetheless these structural changes dramatically decreased endoplasmic reticulum (ER)‐exit and plasma membrane localization of FLA4, with *N‐*glycosylation acting at the level of ER‐exit and *O‐*glycosylation influencing post‐secretory fate. We show that FLA4 acts predominantly by molecular interactions involving its carboxy‐proximal fasciclin 1 domain and that its amino‐proximal fasciclin 1 domain is required for stabilization of plasma membrane localization. FLA4 functions as a soluble glycoprotein via its carboxy‐proximal Fas1 domain and its normal cellular trafficking depends on *N‐* and *O‐*glycosylation.

## Significance Statement

FLA4, a protein involved in root growth, salt tolerance and seed coat mucilage development consists of two fasciclin domains, multiple predicted glycosylation sites and a GPI‐anchoring motif. The functional significance of these features is elusive. Our structural functional dissection shows that the carboxy terminal fasciclin domain is crucial for FLA4 function while the amino‐terminal fasciclin domain, GPI‐anchoring *N‐*linked and *O‐*linked glycosylation are important for intracellular trafficking but not for function.

## Introduction

Arabinogalactan proteins (AGPs) are a large protein family in plants implicated with many biological functions (Seifert and Roberts, 2007; Ellis *et al*., 2010). The subfamily of fasciclin‐like AGPs (FLAs) contains one or two fasciclin 1 (Fas1) domains and several *N‐*glycosylation sites, one or two proline‐rich (PR) domains believed to be *O‐*glycosylated and in most cases a signal for glycosylphosphatidylinositol (GPI) anchoring (Johnson *et al*., 2003). The functional significance of the various Fas1 domains and post‐translational modifications is presently elusive. After fasciclin I was identified in axon fascicles of insects (Bastiani *et al*., 1987; Snow *et al*., 1988; Zinn *et al*., 1988), proteins carrying the fasciclin 1 (Fas1) domain were found in animals, plants, fungi, eubacteria and archaea, and were suggested to act at the interface between the extracellular matrix (ECM) and intracellular signalling (Elkins *et al*., 1990; Thapa *et al*., 2007; Harris and Weigel, 2008; Kim *et al*., 2012; Ghatak *et al*., 2014). The four human Fas1 proteins function in cell to ECM adhesion, as ECM structural elements, in glucosaminoglycan serum clearance, as paracrine signals and in signal perception and they interact with ECM receptors of the integrin family (Kim *et al*., 2000; Harris *et al*., 2008; Zhou *et al*., 2015; Bonnet *et al*., 2016). The function of Fas1 proteins in plants is exclusively understood at the genetic level. The 21 *Arabidopsis thaliana FLA* loci were grouped based on domain organization and sequence similarity (Schultz *et al*., 2000, 2002; Johnson *et al*., 2003). *AtFLA11* and *AtFLA12* and their putative orthologues from other plant species, were implicated with stem mechanical properties and growth, acting either indirectly as regulators or in a direct structural role in secondary cell walls (Li *et al*., 2010a,b; MacMillan *et al*., 2010, 2015; Huang *et al*., 2013; Wang *et al*., 2015). Another single Fas1 domain locus, *AtFLA3*, was essential for microspore formation (Li *et al*., 2010a,b). The tandem Fas1 locus *AtFLA1* played a role in shoot regeneration (Johnson *et al*., 2011). Finally, the tandem Fas1 protein AtFLA4*,* here just named FLA4, encoded by the *SALT‐OVERLY SENSITIVE 5* (*SOS5*) locus, was implicated with a multitude of genetic functions and pathways such as root elongation, salt stress tolerance, ABA and ACC signalling, and seed coat mucilage composition, possibly acting in a linear genetic pathway with the leucine‐rich repeat receptor‐like kinase loci (LRR‐RLK) *AtFEI1* and *AtFEI2* (Shi *et al*., 2003; Xu *et al*., 2008; Harpaz‐Saad *et al*., 2011; Griffiths *et al*., 2014, 2016; Seifert *et al*., 2014). Because of its readily observable root growth phenotype and its expression in root tissue *FLA4* is an attractive model to genetically dissect the relevance of various structural features shared between most FLAs. Specifically, it is presently unclear why many FLAs, including FLA4, have two Fas1 domains in tandem. The *sos5‐1* allele*,* contains a mis‐sense mutation in the carboxy‐proximal Fas1 domain, here named Fas1‐2, and showed a recessive phenotype that is apparently identical to the loss of function allele *sos5‐2* (Xu *et al*., 2008) (this study). This observation supported the essentiality of the Fas1‐2 domain, however, the role of the amino‐proximal Fas1 domain (Fas1‐1) in tandem Fas1 FLAs remains to be investigated. Many FLAs are GPI‐anchored, a feature they share with Fas1 proteins from insects (Hortsch and Goodman, 1990) and fungi (Miyazaki *et al*., 2007; Liu *et al*., 2009). GPI‐anchors were implicated with facilitating the passage of GPI‐anchored proteins (GPI‐APs) through the secretory pathway (Kinoshita *et al*., 2013) and mediating their association to lipid rafts and membrane nanodomains (Malinsky *et al*., 2013; Tapken and Murphy, 2015) or larger subdomains of the plasma membrane (Zurzolo and Simons, 2016). The GPI‐anchor can be cleaved from its protein by endogenous GPI‐specific phospholipases or other enzymes (Fujihara and Ikawa, 2016) releasing the protein into the extracellular space. Localization of functional FLAs *in planta* and the functional role of the GPI‐modification signal are unclear. Based on co‐precipitation of FLAs with AGPs and the β‐glucosyl Yariv (β‐GlcY) reagent (Yariv *et al*., 1962; Kitazawa *et al*., 2013; Paulsen *et al*., 2014) and the presence of PR regions in their protein sequence it is assumed that FLAs contain AGP‐like *O‐*glycans (Schultz *et al*., 2000; Johnson *et al*., 2003; Showalter *et al*., 2010), however, there is no direct evidence for this hypothesis. Moreover, it is unknown what role AG‐decoration might play for FLAs but there is genetic evidence that *O‐*glycosylation itself might be crucial for the function of FLAs. A double mutant lacking the Hyp *O‐*galactosyl transferase genes *AtGalT2* and *AtGalT5* shared salt hypersensitivity of and behaved non‐additively with *sos5*, suggesting that Hyp‐galactosylation of AGPs, although not necessarily of FLA4 itself, might be essential for *FLA4* function (Basu *et al*., 2016). Clear evidence that FLA4 contains an AG‐glycan and how *O‐*glycans might be either directly or indirectly important for FLA4 function could further elaborate this intriguing hypothesis. More generally, it would be important to first obtain evidence if and how the presence and abundance of *O‐*glycosylation influences the function, localization and protein properties of FLAs. In addition to *O‐*glycosylation, FLAs are also thought to be *N‐*glycosylated. The position of *N‐*glycosylation sites is quite reliably predicted from the sequence motif NXT/S, suggesting that in FLAs, the Fas1 domains but not the PR domains contain *N‐*glycans (Schultz *et al*., 2002; Johnson *et al*., 2003). *N‐*glycans are co‐translationally attached and are important for the correct folding of proteins in the endoplasmic reticulum (ER) and for subcellular targeting (Strasser, 2014). Incorrectly folded secretory and transmembrane proteins are recognised in an *N‐*glycan dependent fashion and are proteolytically removed in the conserved ER‐associated degradation (ERAD) pathway (Hüttner and Strasser, 2012). However, as a starting point to investigate the role of *N‐*glycosylation for FLAs, it will be important to identify conserved *N‐*glycans in the Fas1 domain and assess their genetic function.

In the present study, we elucidated the functional significance of the complex structure of FLAs by genetically dissecting structural features of FLA4. We used FLA4 fluorescent protein fusions and structural variants thereof to study the localization, *in vivo* modification and function of this tandem Fas1 protein. We conclude that the secretion of the Fas1‐2 domain is sufficient for *FLA4* function and that this function is supported by domain duplication, GPI‐anchoring and protein glycosylation.

## Results

### Functional FLA4‐citrin is mainly localized at the plasma membrane

**Figure 1:**
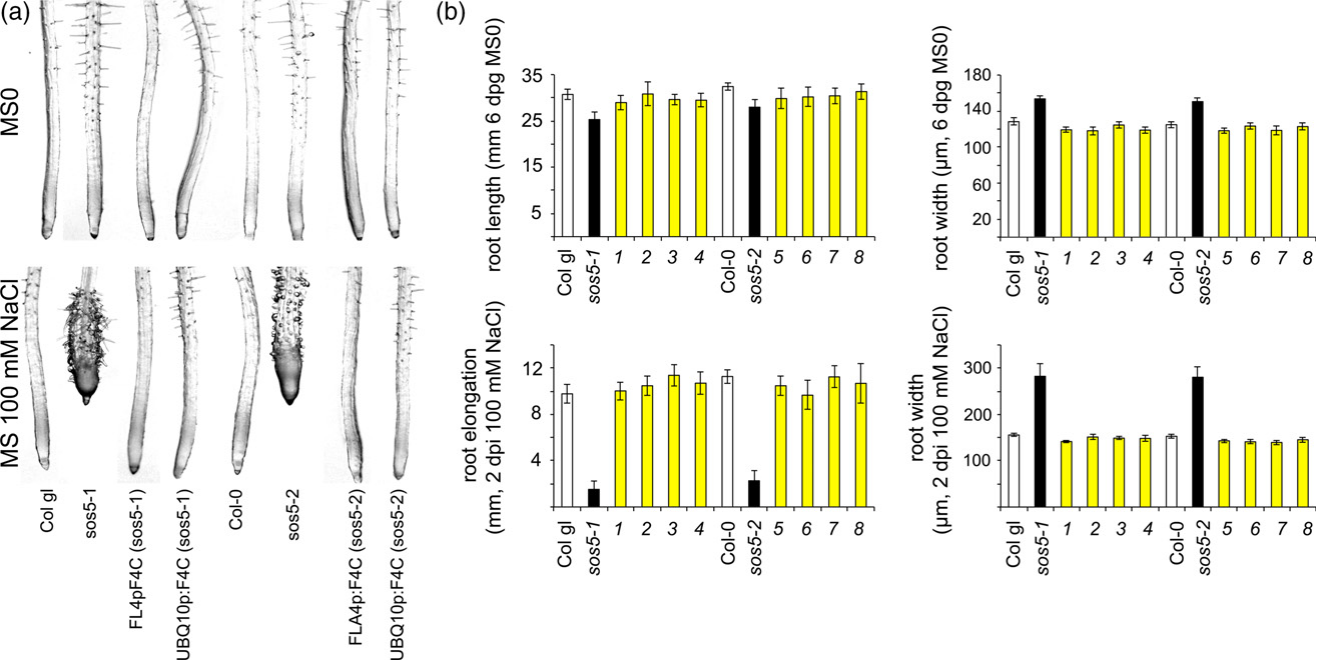
FLA4‐citrin supports normal root growth and salt tolerance in the *sos5‐1* and *sos5‐2* mutant background. (a) Phenotype of 6‐day‐old seedlings growing on standard medium without (MS0) or with 100 mm NaCl (2 dpi) (b) Root length and thickness on MS0 for 5–6 days and root width and elongation growth for 2 days on 100 mm NaCl containing medium. The F4C transgene driven by the FLA4 or the UBQ10 promoter expressed in *sos5‐1* (Col gl) and the *sos5‐2* (Col‐0/Col gl) mutant background show a wild‐type like growth phenotype. The transgenic lines (yellow bars) used for this figure were 1: *FLA4*:*F4C*(4) (*sos5‐1*), 2: *FLA4*:*F4C*(7) (*sos5‐1*), 3: *UBQ10*:*F4C*(5) (*sos5‐1*), 4: *UBQ10*:*F4C*(6) (*sos5‐1*), 5: *FLA4*:*F4C*(4) (*sos5‐2*), 6: *FLA4*:*F4C*(7) (*sos5‐2*), 7:*UBQ10*:*F4C*(3) (*sos5‐2*), 8:*UBQ10*:*F4C*(4) (*sos5‐2*). Error bars indicate the 5% confidence interval.

We generated FLA4‐citrin (F4C), an in‐frame fusion between the predicted FLA4 signal peptide fused to the pH‐stable yellow fluorescent protein monomeric citrin (Shaner *et al*., 2005) followed by the FLA4 coding region including the predicted GPI‐signal (Figure S1), driven by various promoters and transformed *sos5* mutant plants with the constructs. On MS0 standard media both *sos5* alleles showed decreased root length and significantly increased root thickness compared to their respective wild‐type background and after transfer to medium containing 100 mm NaCl, underwent dramatic radial swelling and reduction of elongation (Figure 1) as previously described (Shi *et al*., 2003). Both mutant alleles *sos5‐1* and *sos5‐2*, had an identical effect on root growth. Expression of F4C under the control of either the *FLA4* or the *UBQ10* promoter region in *sos5* mutants, reverted root length and thickness to wild‐type dimensions (Figure 1a, b). Hence F4C fulfils the role of endogenous *FLA4* in root growth and salt tolerance.

**Figure 2:**
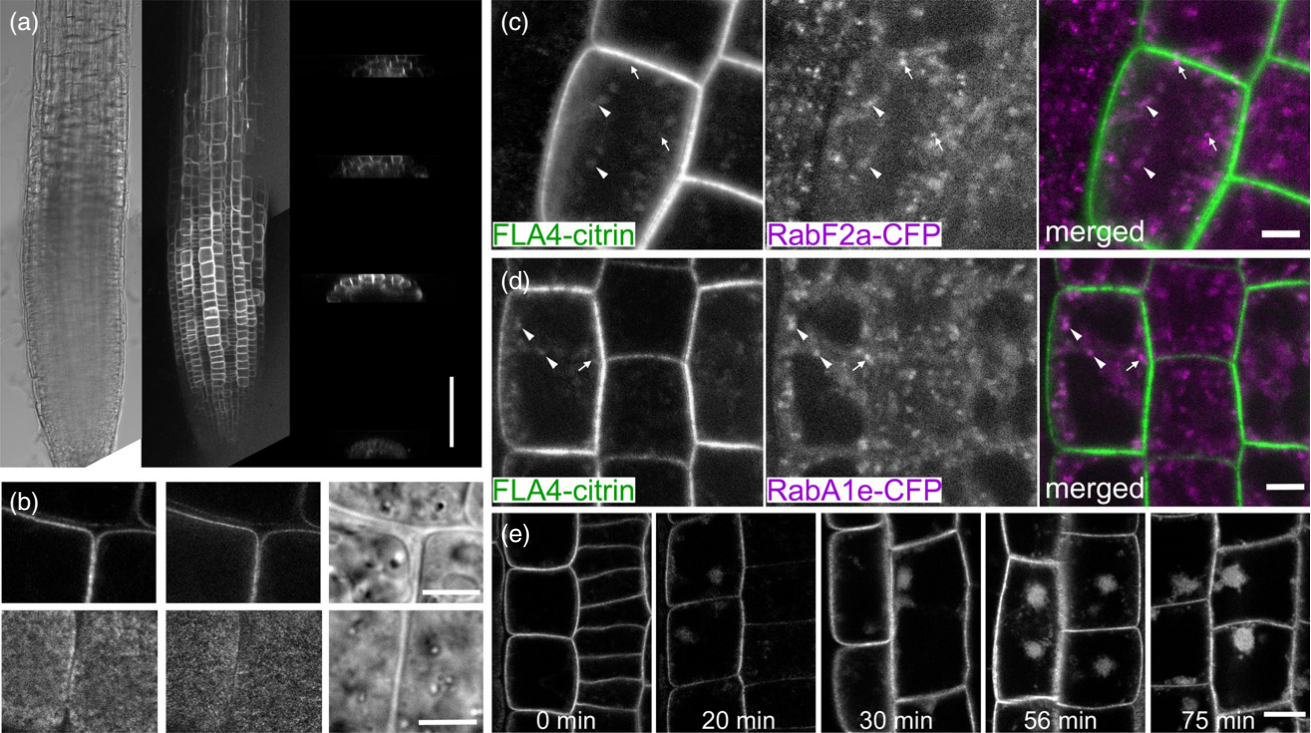
Expression and localization of F4C in roots. (a) Expression of FLA4‐citrin in the root meristem and early elongation zone. Transverse x,z‐projections through the (from bottom) quiescent center, cell division‐, transition‐ and elongation zone. The construct is maximally expressed in the epidermis of the division zone. During maturation the epidermal expression decreases and the expression in more central cell layers increases (scale bar in (a) = 100 μm). (b) High resolution conventional confocal (left) and STED confocal (center) micrograph taken in the cell division zone (scale bar in (b) = 5 μm). (c, d) (c) Partial overlap between FLA4‐citrin and RabF2a‐cerulean or (d) RabA1e‐cerulean in diffuse intracellular compartments (arrowheads) but not in the brightest RabF2a‐cerulean punctae (arrows). Scale bars in (c) and (d) = 5 μm. (e) Treatment with 25 μM brefeldin A for different durations (scale bar in (e) = 10 μm).

Confocal microscopy of seedlings expressing *FLA4*:*F4C*, showed that the transgene was expressed throughout the primary root (Figure 2). The expression reached a local maximum in the late cell division zone in the epidermis, shortly before cells underwent rapid elongation. The cortex and underlying cell layers in the meristematic region expressed the transgene only at a low level. As cells entered the zone of rapid elongation and subsequently the cell differentiation zone, signal intensity in the epidermal layer gradually decreased with a concomitant increase in the cortical layer (Figure 2a, insets). The protein predominantly localized to the cell periphery in a thin line at the cell surface (Figure 2b, compare conventional CLSM and STED left versus center panel). Tangential optical sections of epidermal cells revealed sub‐optical resolution punctae (Figure 2b bottom panel, limit of STED resolution was approximately 80 nm). In standard CLSM mode, at high laser intensity an intracellular signal was seen in addition to the saturated cell‐surface signal. The intracellular F4C signal partially co‐localized with RabF2a‐cerulean (Figure 2c) and with RabA1e‐cerulean (Figure 2d), markers for late and recycling endosomal compartments, respectively (Geldner *et al*., 2009). In both cases, co‐localization occurred in relatively diffuse areas (arrowheads in Figure 2c, d) but not in Rab‐cerulean punctae (arrows in Figure 2c, d). Co‐localization with these two markers suggested a certain degree of endocytosis of F4C. To confirm this possibility we tested the effect of brefeldin A (BFA) which inhibits recycling of endocytosed cargo from the endosome to the plasma membrane and leads to the accumulation of recycling endosomes to approximately 5 μm large intracellular structures, termed BFA bodies (Geldner *et al*., 2003; Naramoto *et al*., 2014). F4C localization was highly sensitive to BFA treatment. After 20 min of 25 μm BFA exposure, F4C localization in BFA bodies became apparent (Figure 2e). After prolonged exposure to the drug, the localization of F4C in BFA bodies predominated over membrane localization. In brief, F4C is mostly localized at the cell surface. Co‐localization of F4C with RabF2a and RabA1e as well as sensitivity to BFA both suggest that FLA4 undergoes endocytosis and is recycled to the plasma membrane in a BFA sensitive manner.

**Figure 3:**
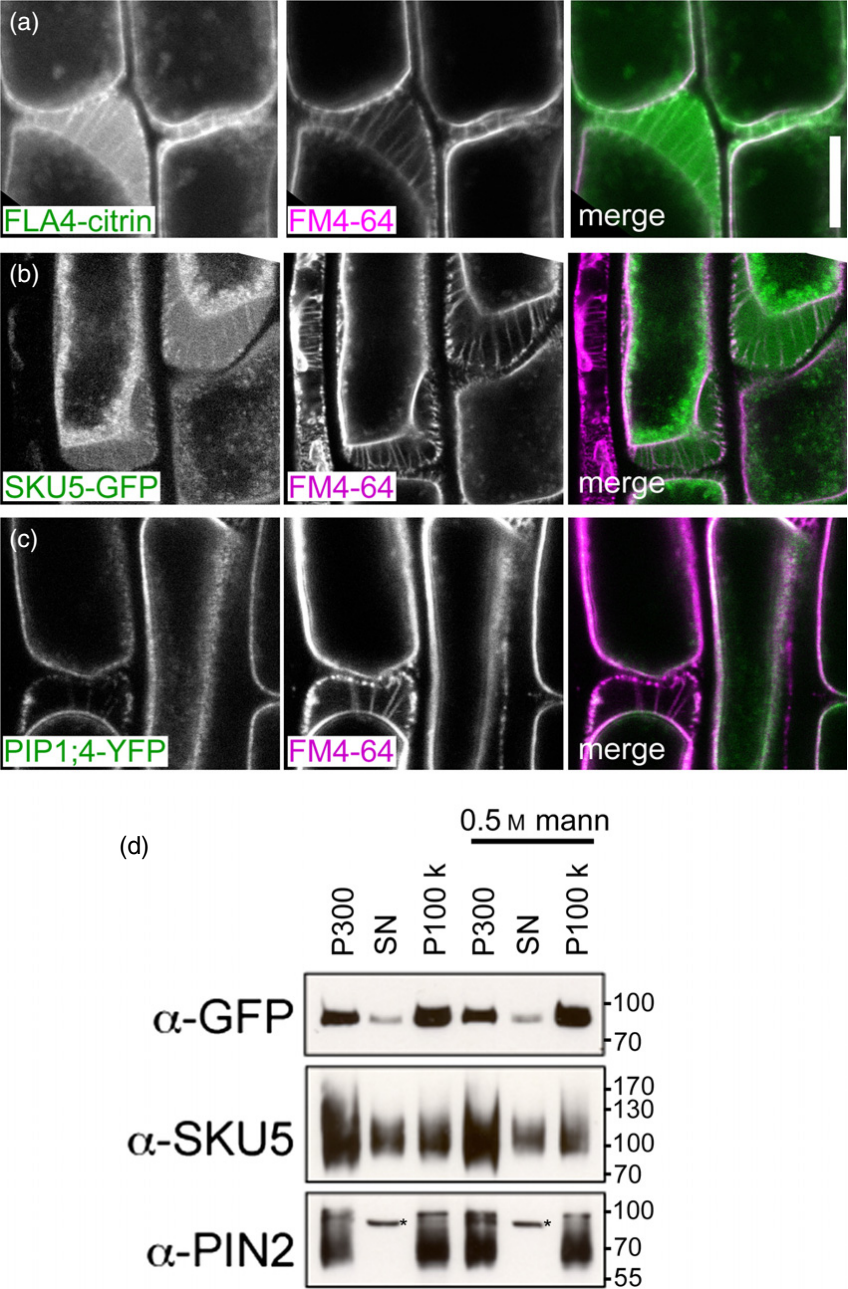
FLA4‐citrin is localized both at the plasma membrane and in the apoplast. (a) FLA4‐citrin and FM4‐64. (b) SKU5‐GFP and FM4‐64. (c) PIP1;4‐YFP. (d) Equivalent proportions (percentage of total) of the pellet after centrifugation at 300 ***g*** (P300) and the supernatant (SN) and pellet (P100k) after subsequent 100 000 ***g*** centrifugation, were loaded on gels and probed with antibodies against GFP, SKU5 and PIN2. The extraction was replicated with material previously exposed to partial plasmolysis by 500 mm mannitol. The asterisk on the blot exposed to a‐PIN2 antiserum indicates an unspecific band as previously noted (Abas *et al*., 2006).

To distinguish between plasma membrane attachment and free apoplastic localization we induced partial plasmolysis using 0.5 m mannitol (Figure 3). In plasmolyzing cells in the root elongation zone, F4C co‐localized with the styryl dye FM4‐64 (Vida and Emr, 1995) at the plasma membrane and in Hechtian strands that remained attached to the cell wall (Figure 3a) and it was localized diffusely in the apoplastic space between the plasma membrane and the cell wall. A similar dual apoplastic and plasma membrane localization was displayed by the GPI‐anchored SKU5‐GFP (Sedbrook *et al*., 2002) (Figure 3c), while the integral membrane protein PIP1;4‐cerulean (Geldner *et al*., 2009) was exclusively membrane localized (Figure 3d). To estimate the overall degree of association of F4C to membranes and the cell wall, we fractionated extracts of roots expressing F4C into a fast sedimenting fraction (P300, ‘cell wall fraction’) a slowly sedimenting fraction (P100k, microsomes) and a soluble fraction (SN). The protein blots were sequentially probed with antibodies for GFP, for SKU5 (Sedbrook *et al*., 2002) and the integral membrane protein PIN2 (Abas *et al*., 2006). F4C was mostly detected in microsomes and to a lesser extent in the ‘cell wall’, with a much lesser contribution from the soluble fraction. By contrast, the SKU5 signal mostly partitioned to the cell wall fraction with the soluble and the microsomal fractions making approximately equal contributions. PIN2, conversely, was present in the ‘cell wall’ and in the microsomal fraction in equal proportions (Figure 3d). This indicated that both putative GPI‐anchored proteins were partially bound to and partially released from the plasma membrane, albeit in an apparently different proportion and that, compared to SKU5 and PIN2, the cell wall association of F4C was only moderate. To test whether short hyperosmotic shock as applied during the plasmolysis experiments, might alter the partitioning of F4C e.g. by activation of endogenous lipases, we also extracted protein from roots that were pre‐exposed to 0.5 M mannitol. However, the partitioning of F4C, SKU5 and PIN2 was not affected by this treatment (Figure 3d). In summary, *F4C* functionally complements endogenous *FLA4* in its role in root growth and salt tolerance. The reporter protein is primarily anchored to the plasma membrane from where it is endocytosed or released to the apoplast.

### FLA4‐citrin can function without its GPI‐modification signal

**Figure 4:**
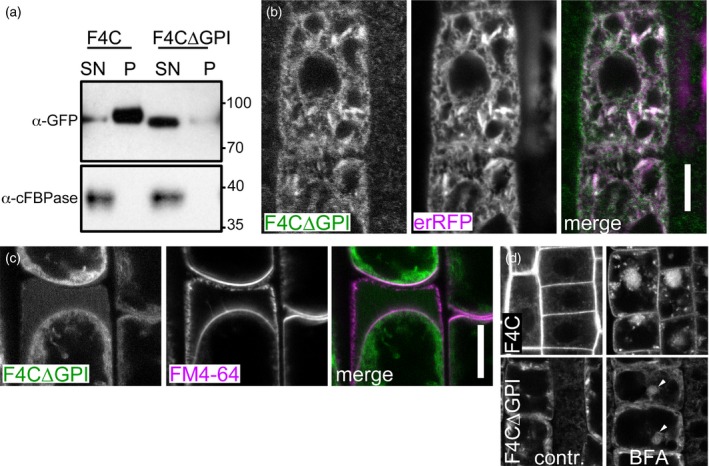
Localization FLA4‐citrin∆GPI. (a) Requirement of the C‐terminal region for membrane association. Transgenic lines expression full‐length FLA4‐citrin (F4C) or C‐terminally truncated F4C (F4C∆GPI) are separated into high‐speed (100 000 ***g***) supernatant (SN) and pellet (P). Cytosolic fructose‐1,6,‐bisphisphatase (cFBPase) is only detected in the soluble fraction. (b) F4C∆GPI co‐localizes with ER‐targeted RFP (erRFP). (c) Plasmolysis of full‐length and FLA4‐citrin∆GPI indicates residual secretion but no plasma membrane localization of F4C∆GPI (scale bar for (c, d) = 10 μm). (d) After brefeldin A (BFA) treatment F4C∆GPI is partially localized to BFA body‐like structures (arrowheads).

A plausible mechanism of dual localization of F4C at the plasma membrane and in the apoplast is by means of GPI‐anchoring. Instability of the GFP epitope towards GPI‐PLC digestion and apparent release of the protein from the membrane fraction under control conditions precluded unambiguous enzymatic demonstration of GPI‐modification (Figure S2). To test whether the C‐terminus that is predicted to act as GPI‐modification signal, is required for the membrane attachment of F4C we transformed plants with the *F4C∆GPI* construct that lacked the 25 aminoacid residues at the FLA4 C‐terminus (Figure 4). In contrast to the wild‐type fusion protein, the C‐terminally truncated construct partitioned to the soluble fraction supporting the requirement of the C‐terminal domain for membrane attachment (Figure 4a). This was confirmed at the microscopic level, where F4C∆GPI failed to label the plasma membrane but was in a mostly intracellular localization where it co‐localized with ER‐retained red fluorescent protein (erRFP) (Gallavotti *et al*., 2008) (Figure 4b). Co‐localization was apparently complete including an intracellular mesh and fusiform ER bodies (Hawes *et al*., 2001; Nakano *et al*., 2014). Upon plasmolysis, most of the F4C∆GPI derived signal was retained in the cytoplasm while the extracellular space was filled with a faint diffuse signal but neither plasma membrane nor Hechtian strands were labelled by F4C∆GPI (Figure 4c). This suggested that the protein lacking the GPI‐anchor signal, was partially secreted but was not membrane associated. Interestingly, exposure to BFA triggered the recruitment of F4C∆GPI into BFA bodies, albeit at a lower intensity compared to the full‐length construct (arrowheads, Figure 4d). Surprisingly, despite its importance for membrane attachment and ER‐exit, the GPI‐modification signal of FLA4, was not required for genetic function as indicated by normal root growth of *F4C∆GPI* (*sos5‐1*) and *F4C∆GPI* (*sos5‐2*) plants on medium with and without 100 mm NaCl (Figure S3). In summary, the FLA4 GPI‐modification signal is required for membrane anchoring, efficient ER‐exit, secretion and plasma membrane localization of F4C, while in the absence of membrane anchoring a low level of secretion and endocytosis takes place and, most importantly, the genetic function in roots remains intact.

### The variable amino‐proximal Fas1 region stabilizes plasma membrane localization but is not essential for FLA4 function in root growth

**Figure 5:**
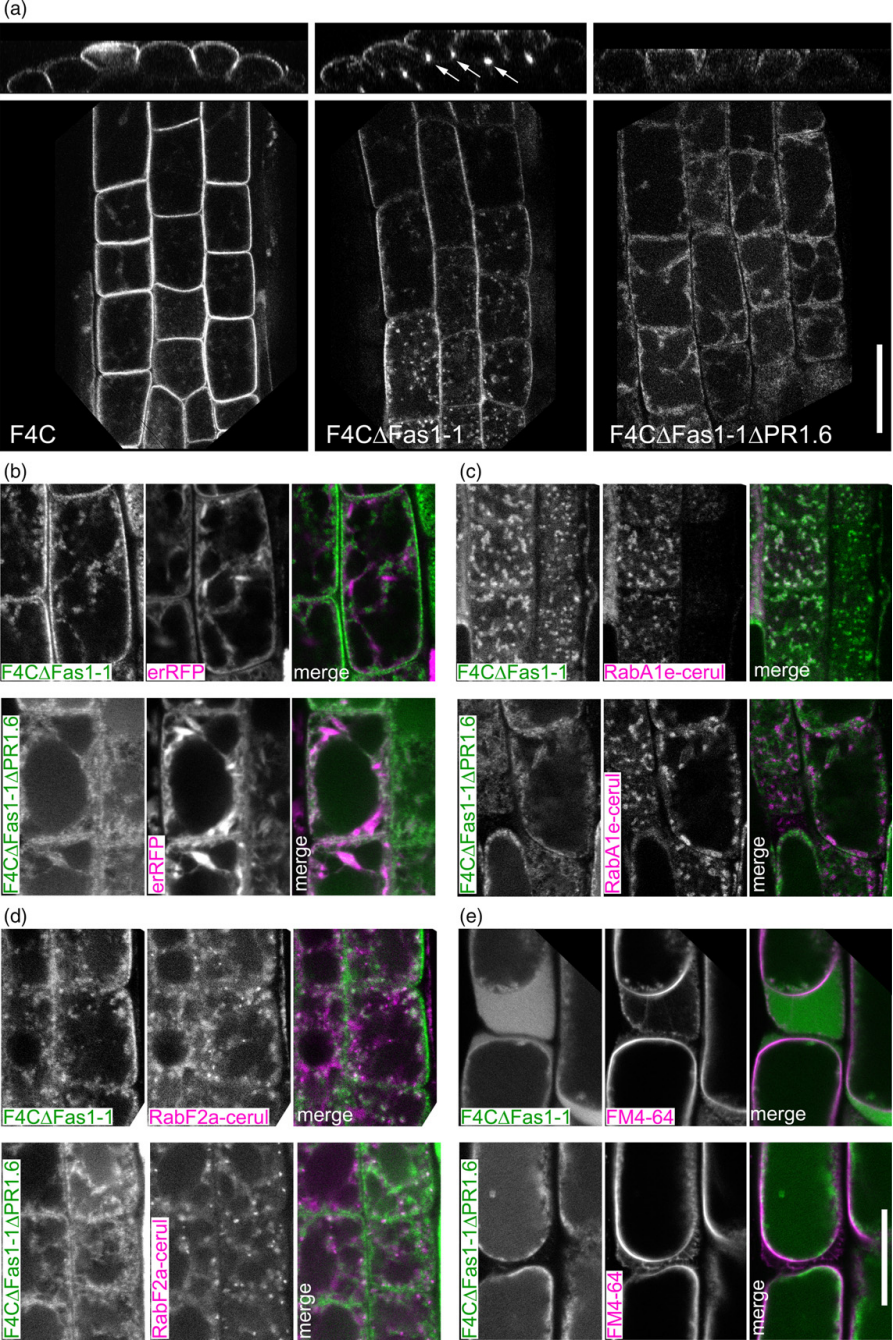
Normal localization of F4C depends on the *N‐*proximal region of the protein. (a) Constructs lacking only the Fas1‐1 region (F4C∆Fas1‐1) or the Fas1‐1 and the entire PR1 and the *N‐*terminus of the Fas1‐2 region (F4C∆Fas1‐1∆PR1.6) are mis‐localized to different compartments. (b) Co‐labelling of F4C∆Fas1‐1 and F4C∆Fas1‐1∆PR1.6 with erRFP. (c) Co‐labelling of F4C∆Fas1‐1 and F4C∆Fas1‐1∆PR1.6 with RabA1e‐cerulean. (d) Co‐labelling of F4C∆Fas1‐1 and F4C∆Fas1‐1∆PR1.6 with RabF2a‐cerulean. (e) Co‐labelling of F4C∆Fas1‐1 and F4C∆Fas1‐1∆PR1.6 with FM4‐64 in partially plasmolyzed cells.

The significance of the tandem Fas1 organization of FLA4 is presently elusive. A protein sequence alignment of putative *FLA4* orthologues from different phyla of flowering plants showed that the Fas1‐2 domain was more conserved than the Fas1‐1 domain (Figure S4). Between the aligned sequences, the Fas1‐1 domain was 22% identical and 44.9% divergent while the Fas1‐2 domain was 55% identical and 20.2% divergent. The evolutionary trend of higher structural divergence in Fas1‐1 compared to Fas1‐2 was confirmed by a survey of naturally occurring sequence polymorphisms among hundreds of *Arabidopsis thaliana* accessions in the 1001 genomes database (http://1001genomes.org/). In the 145 residues of the Fas1‐1 domain the 1001 genomes database yielded 11 non‐synonymous DNA polymorphisms giving rise to eight divergent aminoacid substitutions in the FLA4 protein sequence, while in the Fas1‐2 domain there were only two highly conserved polymorphisms (Figure S5). To experimentally address the functional role of the Fas1‐1 domain for *FLA4* function we generated versions of F4C lacking the Fas1‐1 domain and varying lengths of the central proline‐rich (PR1) domain (Figure S6a). Most constructs lacking the Fas1‐1 and the PR1 domain complemented the *sos5* mutants (Figure S6b). The shortest construct consistently complementing *sos5*,* UBQ*:*F4C∆Fas1‐1∆PR1*.*4*, lacked both the Fas1‐1 and the entire PR1 domain. While 100% of the lines transformed with either the *UBQ*:*F4C∆Fas1‐1∆PR1*.*4* construct or less truncated versions displayed complementation of *sos5* (*n *=* *32 independent transformants), the construct *UBQ*:*F4C∆Fas1‐1∆PR1*.*5* in which four more residues including the predicted *N‐*glycosylation site N207 were deleted, was functional in only 50% of tested transgenic lines (*n *=* *10). Removal of seven more aminoacid residues in the *F4C∆Fas1‐1∆PR1*.*6* construct completely abolished genetic function (Figure S6b). We next assessed whether the Fas1‐1 domain was important for either protein localization or abundance *in vivo*. Like the full‐length fusion protein, F4C∆Fas1‐1 displayed cell peripheral localization and localized to intracellular particles, however, the abundance in intracellular structures was strongly increased in the mutant compared to the wild‐type version (Figure 5). Moreover, the F4C∆Fas1‐1 product very brightly localized to cell corners i.e. junctions between epidermal and cortical cells (Figure 5a, arrows in top panel). The intracellular F4C∆Fas1‐1 particles did not notably overlap with erRFP (Figure 5b) but co‐localized with RabA1e‐cerulean (Figure 5c) and RabF2e‐cerulean (Figure 5d), to a greater degree than the full‐length protein (compare with Figure 2d). By contrast, F4C∆Fas1‐1∆PR1.6 solely resided in an intracellular network (Figure 5a) that co‐localized with erRFP (Figure 5b) but neither with RabA1e‐cerulean (Figure 5c) nor with RabF2e‐cerulean (Figure 5d). Plasmolysis of root cells expressing the truncated constructs showed a bright F4C∆Fas1‐1‐derived signal in the apoplast and less bright labelling of the plasma membrane, while F4C∆Fas1‐1∆PR1.6 remained strictly intracellular (Figure 5e). Treatment with BFA led to the accumulation of F4C∆Fas1‐1 in BFA bodies while the distribution of F4C∆Fas1‐1∆PR1.6 product was unaffected by the drug (Figure S7). The microscopic data suggested that FLA4∆Fas1‐1 efficiently exited the ER, travelled all the way down the secretory pathway to the plasma membrane and the apoplast and either was rapidly endocytosed, accumulating in RabA1e positive recycling endosomes or was shed into the apoplast. By contrast, the localization of F4C∆Fas1‐1∆PR1.6 suggested that the protein was completely retained in the ER. To more precisely determine the region in FLA4∆Fas1‐1 that is responsible for ER‐exit of the amino‐proximally truncated but functional forms of FLA4, we microscopically analysed transformants expressing transgenes F4C∆Fas1‐1∆PR1.1 to F4C∆Fas1‐1∆PR1.5 (Figure S8). In the F4C wild‐type and the F4C∆Fas1‐1 and F4C∆Fas1‐1∆PR1.1 to F4C∆Fas1‐1∆PR1.4 products we observed both plasma membrane and endosomal localization with the mutant lines additionally showing localization in cell corners and a greater proportion of endosomal vs. plasma membrane localization compared with the wild‐type. However, the *F4C∆Fas1‐1∆PR1*.*5* localization mostly resembled the ER‐like pattern of F4C∆Fas1‐1∆PR1.6 and was hardly visible at the cell surface (Figure S8, arrow). In summary, the Fas1‐1 domain was not required for *FLA4* function but was required to stabilize plasma membrane localization of the protein. On the other hand the integrity of the amino‐proximal region of the Fas1‐2 domain, was not only essential for genetic function but also for the exit from the ER. We next investigated the significance of *N‐*glycosylation of FLA4 and specifically of the amino‐proximal region of Fas1‐2.

### A conserved *N‐*glycan at the boundary of Fas1‐2 is required for efficient secretion but not for genetic function

**Figure 6:**
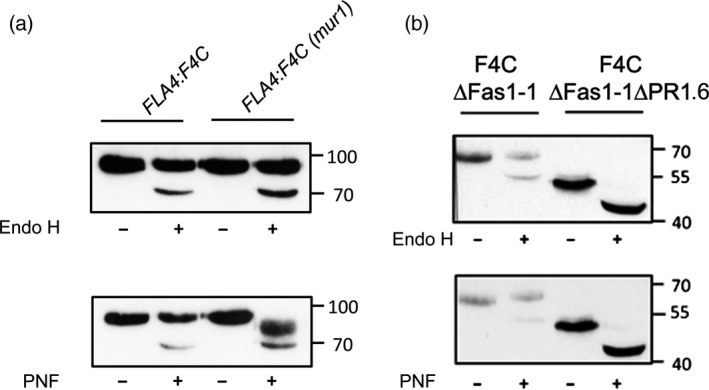
*N‐*glycosylation of F4C. (a) Treatment of oligomannosidic *N‐*glycans by Endo H and release of complex *N‐*glycans by PNF in the *MUR1* wild‐type and the GDP‐L‐fucose defective *mur1‐2* background. In mur1‐2 F4C becomes fully sensitive to PNF indicating fucosylation and additional *N‐*glycan processing. (b) The F4C∆Fas1‐1 product is only partially sensitive to Endo‐H and PNF while the ER‐retained F4C∆Fas1‐1∆PR1.6 product is fully sensitive to both enzymes indicating that it exclusively contains oligomannosidic *N‐*glycans.

**Figure 7:**
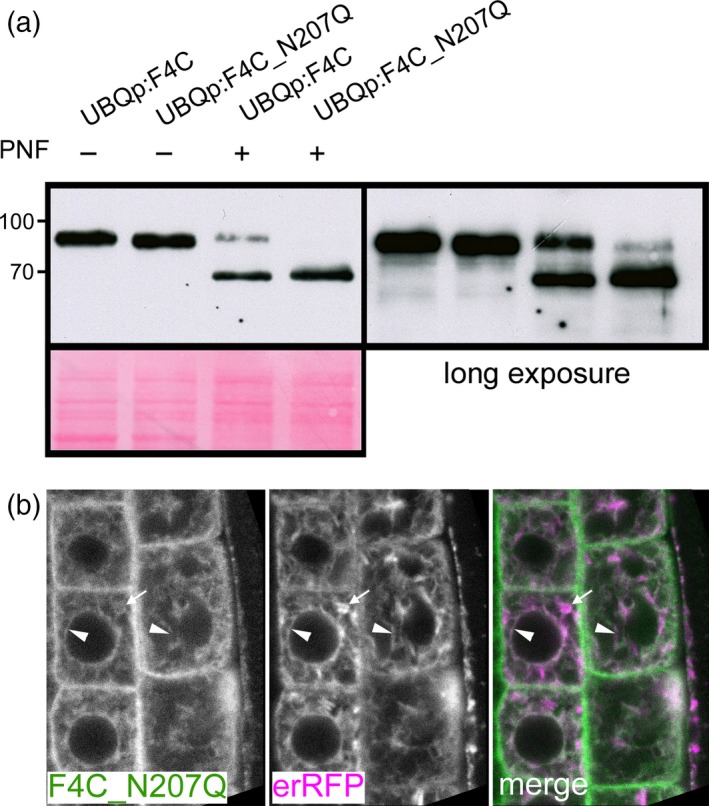
The conserved *N‐*glycosylation site N207 is required for normal endoplasmic reticulum (ER) to Golgi transit. (a) The substitution mutant F4C_N207Q shows strongly increased sensitivity to PNF indicating an increased ratio of oligomannosidic to fucosylated complex *N‐*glycans. A minor residual fraction of PNF resistant product is only detected when blots are over‐exposed. (b) F4C_N207Q co‐localizes with reticulate ER structures (arrowheads) but does not accumulate in fusiform ER bodies (arrows).

FLA4 contains eight predicted *N‐*glycosylation sites and might be the most highly *N‐*glycosylated *Arabidopsis thaliana* FLA (Table S1). To experimentally confirm the presence of *N‐*glycans on F4C we used *N‐*glycan specific glycosidases (Figure 6). Endoglycosidase H (Endo‐H) cleaves the glycosidic linkage between the two core *N‐*acetyl glucosamine (GlcNAc) residues in oligomannosidic but not in complex *N‐*glycans. Treatment of F4C extract with Endo‐H induced a molecular weight shift of a minor fraction of F4C resulting in a weak sharp band of approximately 66 kDa (Figure 6a). Because the untreated F4C smeared between 86 and 96 kDa, we calculated a molecular weight shift between 20 and 30 kDa. This Endo H sensitive fraction represented F4C carrying oligomannosidic *N‐*glycans en route to the Golgi. Peptide *N‐*glycosidase F (PNGase F) cleaves the glycosidic links between the innermost GlcNAc residue and asparagine of *N‐*glycoproteins in both oligomannosidic and complex *N‐*glycans, however, the enzyme is blocked by α(1–3) fucosylation at the innermost GlcNAc of complex *N‐*glycans. Treatment of F4C with PNGase F resulted in an electrophoretic pattern identical to the one seen after Endo‐H digestion. This suggested that most complex *N‐*glycans on F4C might be α(1–3) fucosylated. To test this hypothesis we crossed *FLA4*:*F4C* with the GDP‐L‐fucose biosynthesis defective *mur1‐2* mutant (Reiter *et al*., 1997). In the *mur1‐2* background, F4C was fully sensitive to PNGase F as seen by the complete disappearance of product at 91 kDa (Figure 6a). The resulting F4C signal was found in a minor sharp band at 66 kDa, exactly as seen in the *MUR1* WT background and in a novel fraction that smeared between 73 and 83 kDa with its maximum at around 78 kDa. Therefore, α(1–3) fucosylated complex *N‐*glycans contributed around 13 kDa to the major fraction of F4C. Variably sized additional post‐translational modifications contributing 7–17 kDa were only present on the Endo‐H insensitive F4C fraction. We next tested the *N‐*glycosylation of two amino‐proximally truncated versions of F4C namely F4C∆Fas1‐1 and F4C∆Fas1‐1∆PR1.6. Like full‐length F4C, a small fraction of F4C∆Fas1‐1 experienced a downward shift in apparent molecular weight after digestion with Endo‐H and PNGaseF (Figure 6b), suggesting that F4C∆Fas1‐1 and the wild‐type protein were *N‐*glycosylated in the ER and underwent *N‐*glycan remodelling in the Golgi at a similar rate. By contrast*,* digestion with both Endo‐H and PNGase F of F4C∆Fas1‐1∆PR1.6 led to the full downward shift of the *entire* protein (Figure 6b), indicating that F4C∆Fas1‐1∆PR1.6 was exclusively decorated with oligomannosidic *N‐*glycans. Consistent with its microscopic localization this suggested that F4C∆Fas1‐1∆PR1.6 was fully retained in the ER. To assess which *N‐*glycosylation sites were most highly conserved in Fas1 domains of FLAs, we aligned the individual Fas‐1 domains of all *Arabidopsis thaliana* FLAs (Figure S9). While several Fas1 domains contained predicted *N‐*glycans directly adjacent to their H1 domains (Fas1‐1 in FLA15 to ‐18, FLA21) or H2 domains (FLA3, ‐5, ‐14 and Fas1‐1 in FLA1, ‐2, ‐4, ‐8 and ‐10) the most conserved position for *N‐*glycosylation in Fas1 domains was 31–39 residues upstream the H1 domain. We detected this predicted glycosylation site in every single Fas1 domain FLA except FLA19 and in every tandem Fas1 domain FLA except FLA15 to FLA18, FLA20 and Fas1‐2 in FLA2. (Table S1 and Figure S9). As the region containing the predicted glycosylation site at N207 was crucial for secretion and also might contribute to the function of FLA4 (previous section), we assessed the significance of *N‐*glycosylation at residue N207 in the Fas1‐2 domain by replacing it by glutamine (Q) in the full‐length fusion protein to generate the transgene *UBQ*:*F4C_N207Q*. The relative abundance of the resulting protein was comparable to the wild‐type, suggesting that this post‐translational modification was not crucial for either protein stability or turnover *in vivo* (Figure 7). However, the proportion of PNGaseF sensitive and ‐insensitive protein was shifted towards relatively more PNGaseF sensitive (Figure 7a). This suggested that F4C_N207Q might either be retained in the ER or not be efficiently modified by Golgi localized *N‐*glycan remodelling enzymes. In agreement with the former possibility, F4C_N207Q displayed predominant co‐localization with the ER marker erRFP with only a minor proportion of F4CN207 localized at the cell periphery (Figure 7b). Despite the dramatic influence of this highly conserved glycosylation site on protein trafficking, the *F4C_N207Q* transgene displayed genetic complementation comparable to wild‐type controls (Figure S10). Taken together, we show that F4C is highly *N‐*glycosylated and that the conserved *N‐*glycan abutting the Fas1‐2 domain of FLA4 plays a crucial role for ER‐exit but is not essential for function.

### FLA4 carries AGP‐specific *O‐*glycan epitopes

**Figure 8:**
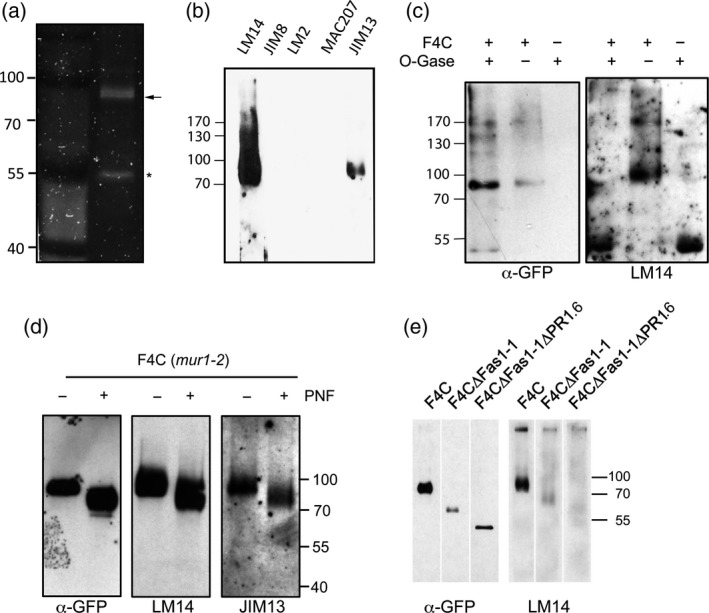
F4C carries AGP‐associated carbohydrate epitopes. (a) A SDS‐PAGE gel of affinity‐purified F4C shows a protein doublet (arrow) at 91 kDa and a single protein band (asterisk) at around 53 kDa. (b) Out of a variety of carbohydrate‐specific monoclonal antibodies only LM14 and JIM13 react with affinity‐purified F4C. (c) Treatment of affinity‐purified F4C with *O‐*glycosidases removes reactivity with LM14. (d) Both AGP antibodies react with heterogeneous fraction that is PNF sensitive only in the *mur1‐2* background but not with the minor fraction that is generally PNF sensitive. (e) LM14 reacts with full‐length F4C and with truncated F4∆Fas1‐1 but not with the ER‐retained F4C∆Fas1‐1∆PR1.6.

There is so far no direct evidence that FLAs contain AGP‐like *O‐*glycans and no sequence motif precisely predicts (nor excludes) proline hydroxylation and *O‐*galactosylation. Classical AGPs contain (XO)_≥2_ repeats in their peptide sequence with X typically a Ser, Thr or Ala residue preceding hydroxyproline (Kieliszewski, 2001) and in all *bona fide* AGPs there are regions in the protein sequence that contain a high combined frequency of Pro, Ala, Ser and Thr (PAST) (Showalter *et al*., 2010). Although some FLAs such as FLA4 do not contain (XP)_≥2_ repeats, all FLAs contain one or two Pro‐rich domains that display a high local PAST frequency (Johnson *et al*., 2003; Showalter *et al*., 2010). To obtain direct evidence for the presence of AGP‐specific glycans on a functional FLA, we probed purified F4C with AGP‐glycan specific monoclonal antibodies (Figure 8). After a one‐step affinity purification of F4C we detected a protein doublet at around 91 kDa on a denaturing reducing gel stained with SYPRO Ruby (Figure 8a). Mass spectrometry of tryptic peptides of the excised double band revealed FLA4 and YFP as only specific proteins in this fraction (Figure S11). An additional band at 53 kDa corresponded to ribulose bisphosphate carboxylase (Rubisco, AtCg00490) a frequent contaminant due to its great abundance. On protein blots of purified F4C, the AGP‐specific monoclonal antibodies LM14 (Moller *et al*., 2008) and JIM13 (Knox *et al*., 1991) reacted with a band at around 91 kDa after short film exposures and after longer exposures with a high molecular weight smear while monoclonal antibodies specific for other AGP‐glycan and cell wall carbohydrate epitopes did not bind to purified F4C (Figure 8b, see Experimental Procedures for a full description of tested antibodies and references). Even at very long exposures the signal did not extend below 91 kDa. LM14 and JIM13 were previously shown to bind to glycan epitopes on AGPs (Yates *et al*., 1996; Moller *et al*., 2008). To further confirm the glycan nature of the F4C‐decorating epitopes we digested purified F4C with a combination of glycosidases, that specifically hydrolyse glycosidic links in type II AGs (Tsumuraya *et al*., 1990; Konishi *et al*., 2008; Takata *et al*., 2010; Tryfona *et al*., 2010, 2012). After glycosidase treatment the apparent molecular weight of F4C subtly shifted downwards and the LM14 epitope was no longer detected (Figure 8c). To exclude the possibility that the detected AGP epitopes might belong to a contaminating AGP with a molecular weight similar to F4C, we manipulated the molecular weight of F4C in two alternative ways. First we removed the *N‐*glycans. As shown in the previous section the removal of *N‐*glycan of F4C in the *mur1‐2* mutant background generated two fractions, one sharp 66 kDa band and a more heterogeneous fraction smearing between 73 and 83 kDa. Probing PNGase F digested immuno‐purified F4C (*mur1‐2*), only the heterogeneous fraction but not the discrete 66 kDa band reacted with LM14 and JIM13 (Figure 8d). Alternatively, we tested antibody reactivity with F4C∆Fas1‐1 that was normally processed in the secretory pathway and with F4C∆Fas1‐1∆PR1.6 that was retained in the ER. The immuno‐purified F4C∆Fas1‐1 protein reacted with LM14 indicating that this variant is *O‐*glycosylated. By contrast, purified F4C∆Fas1‐1∆PR1.6 did not react with LM14 (Figure 8e). Together with the ER‐localization of F4C∆Fas1‐1∆PR1.6 this result was consistent with the notion that the LM14 epitope was generated in a post ER compartment. Both sets of data supported our claim that the detected *O‐*glycan epitopes were a part of F4C and not a contaminating AGP of similar molecular weight. In brief, we directly demonstrated that F4C contains AGP epitopes that are generated in a post ER compartment.

### Proline‐rich regions are not essential for *O‐*glycan decoration and function of FLA4

**Figure 9:**
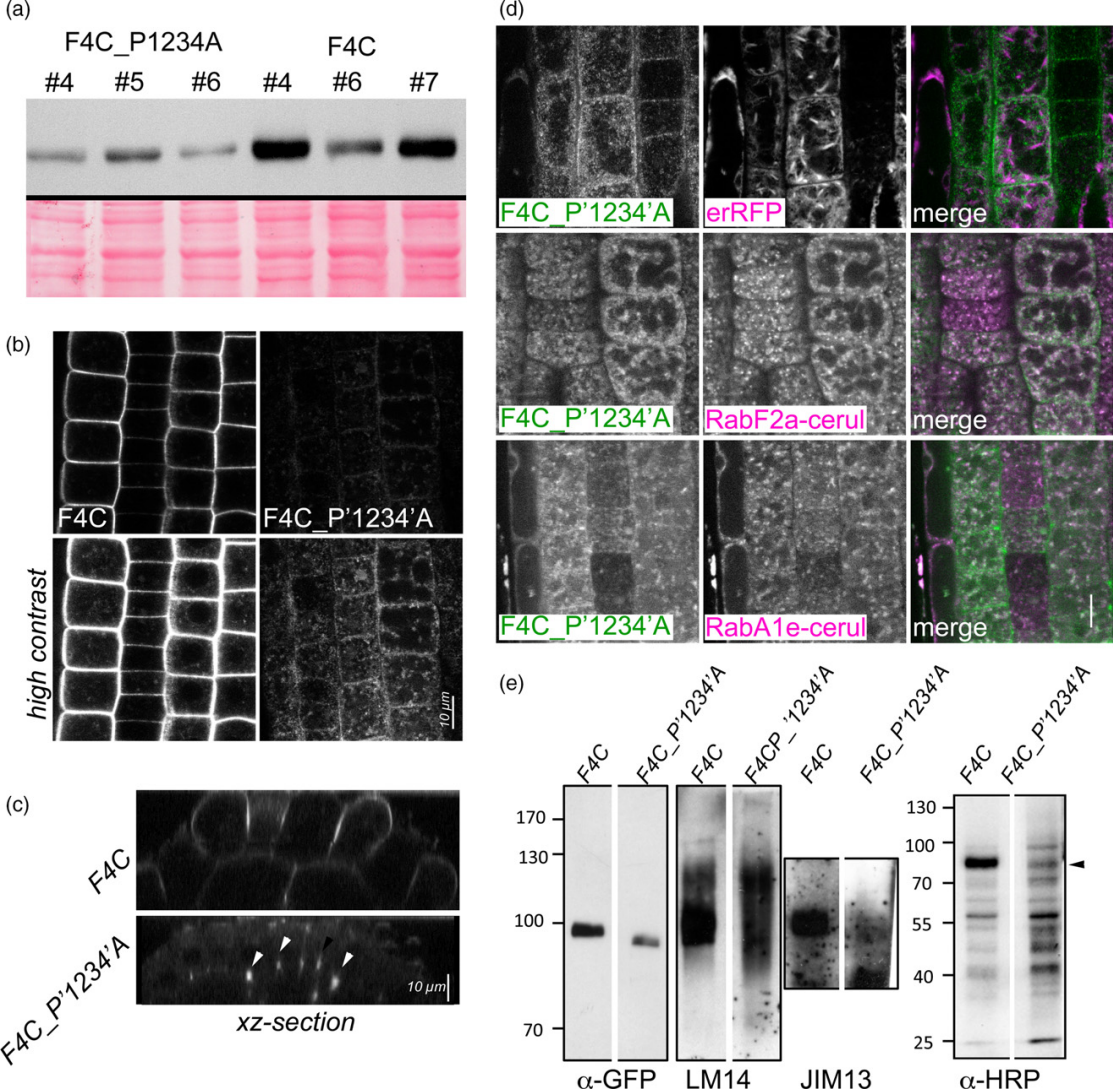
Substitution of Pro residues in Pro‐rich domain affects abundance and localization of F4C. (a) Three independent homozygous transformant lines of FLA4:F4C_P1234A and FLA4:F4C are compared on a western blot. (b) F4C line #4 and F4C:P1234A #5 are compared with identical imaging settings with normal (top panel) and increased contrast (bottom panel). (c) An XZ‐section shows F4C‐P1234A derived signal in cell corners (arrowheads). (d) F4C_P1234A co‐localizes with endosomal markers RabA1e‐cerulean and RabF2a‐cerulean while there is only weak overlap with erRFP. (e) Affinity‐purified F4C_P1234A reacts with LM14 but not with JIM13. Labelling with the complex‐*N‐*glycan reacting HRP antibody shows a strong relative increase of small fragments compared to a size reduced (arrowhead) full‐length fragment.

What is the functional role of the PR domains on FLAs? In FLA4, the PR1 domain contains two clusters of S/TPPP, while the carboxy‐proximal proline‐rich region (PR2) contains two proline doublets (APP and SPP). Comparing putative *FLA4* orthologues we found the PR1 domain not well conserved except for P202, which was placed five positions upstream the *N‐*glycosylation site N207 in all investigated putative FLA4 orthologues (Figure S4). In the PR2 domain, the two proline doublets P368P369 and P389P390 were conserved in all putative *FLA4* orthologues. We addressed the requirement of PR regions for FLA4 glycosylation and function by replacing all clustered Pro residues with Ala in the construct *F4C_P1234A* (Figure 9). On western blots the modified fusion protein migrated as subtly downward shifted band and, comparing the intensities of independent homozygous transformed lines between F4C_P1234A and F4C, the mutants generally produced weaker signals (Figure 9a). Consistently, a subtle reduction in F4C signal intensity was also observed when seedlings were treated with the prolyl‐4‐hydroxylase inhibitor bipyridyl (Figure S12). The reduced abundance of F4C_P1234A compared with F4C appeared more dramatic at the cellular level and the mutant protein localized predominantly to pleomorphic intracellular structures and only to a very minor extent to the plasma membrane (Figure 9b). Similar to amino‐proximal truncation constructs, however, a bright signal emanated from cell corners (arrowheads, Figure 9c). Co‐localization with erRFP was only moderate (Figure 9d, top panel). By contrast, the intracellular fraction of F4C_P1234A largely co‐localized with both endosomal markers RabF2a‐cerulean and RabA1e‐cerulean (Figure 9d, middle and bottom panel, respectively). On SDS‐PAGE gels that were run for a sufficiently long duration we observed a 3 kDa downward shift in molecular weight of purified F4C_P1234A compared with F4C (Figure 9e). LM14 reacted with the Ala to Pro substituted version and the wild‐type fusion protein with similar intensity, however, the smear typically seen at long exposures extended not only to the higher but also the low‐molecular‐weight range compared with the main product that coincided with the GFP epitope tag. By contrast, the signal produced by the JIM13 antibody was strongly reduced in the F4C_P1234A construct (Figure 9e). In our initial efforts to purify F4C_P1234A we had difficulties to detect the protein and had to load relatively large amount of eluate. We roughly assessed the integrity of the purified protein exploiting the decoration of FLA4 with fucosylated complex glycans and the recognition of these glycans by horseradish peroxidase (HRP) antibodies (Wilson *et al*., 1998). The purified wild‐type F4C product was strongly and some lower MW bands were weakly detected by the HRP antibody, suggesting relatively good integrity of the purified protein. By contrast, the anti‐HRP signal at the full molecular weight of the F4C_P1234A variant was strongly reduced in favour of more intense labelling of low‐molecular‐weight fragments. Together these data suggested that the mutated protein might be more prone to degradation *in vivo*. Despite its effect on protein abundance and stability, to our surprise, the construct was genetically functional in our complementation assays (Figure S13) indicating that PR regions were not essential for the function of *FLA4* in root growth. To summarize the observations made in the previous section, substitutions of the most conserved prolines in the PR domains remain inconsequential with respect to genetic role of F4C despite affecting *O‐*glycan epitope reactivity as well as protein abundance, stability and localization.

## Discussion

### FLA4 can act as a low abundance soluble factor in the apoplast

Using the *sos5* root growth and salt tolerance phenotype and previously its seed coat mucilage phenotype (Griffiths *et al*., 2016) we showed that *F4C* functions in the role of *FLA4* and hence reports the native developmental and cellular localization of FLA4. Although we observed variations in intensity between independent lines containing the *FLA4*:*F4C* transgene, the developmental pattern of *F4C* expression in roots remained the same. The maximal expression in the early transition zone between the cell division and the cell elongation zone is suggestive of a role of *FLA4* in the transition from cell division to elongation. It is also consistent with the massive radial swelling of salt‐stressed *sos5* roots in the meristematic zone. The combination of cell fractionation and microscopy suggested that F4C is primarily localized at the plasma membrane and, with lower abundance, in the apoplast and in endosomal compartments. The localization of F4C at the plasma membrane is consistent with a role of FLA4 as co‐receptor of the FEI1/FEI2 LRR‐RLKs as it was previously suggested (Shi *et al*., 2003; Xu *et al*., 2008; Basu *et al*., 2016) where it might act in cell wall sensing (Showalter and Basu, 2016). However, GPI‐anchored FLA4 might also serve as a reservoir at the plasma membrane for protein to be released and to act as a soluble molecule in the apoplast. It was previously suggested that FLA4 might fulfil a structural role for seed coat mucilage adherence based on the observation that F4C was largely localized in the mucilage pocket (Griffiths *et al*., 2016). Although we cannot exclude that a minor fraction of F4C is loosely attached to cell walls of fully turgid cells, there was no evidence for abundant and tight binding of F4C to the cell wall in plasmolyzed cells and the proportion of F4C in the P300 fraction was much lower compared to two other membrane proteins, SKU5 and PIN2 (Figure 3). Hence we conclude that in roots under our standard growth conditions F4C, is more abundant in the plasma membrane compared to the apoplast. The coexistence of membrane bound and soluble forms of F4C, similar to SKU5‐GFP, is best explained by GPI‐anchoring. Although the generally accepted assay for GPI‐anchored proteins, digestion by bacterial GPI‐PLC, did not produce a fully satisfactory result for F4C due to technical reasons (Figure S2), the truncation of the hydrophobic C‐terminal region that is required for GPI‐anchoring caused a redistribution of F4C from the plasma membrane to the ER and a shift of the protein from the microsomal to the soluble fraction (Figure 4a), which suggest that the C‐terminus is required for the efficient plasma membrane localization of FLA4. However, the localization of F4C∆GPI in the apoplast of plasmolyzed cells suggests that a small fraction of the GPI‐less protein is still able to complete its passage through the secretory pathway. Our finding that *FLA4∆GPI* was genetically active is reminiscent of GPI‐less but complementing LORELEI (Li *et al*., 2015; Liu *et al*., 2016). Our result contradicts the hypothesis that FLA4 acts as a GPI‐anchored component of a plasma membrane signalling complex (Basu *et al*., 2016; Showalter and Basu, 2016) or to act as a physical link between the cell wall and nanodomain‐localized signalling complexes as recently suggested (Tapken and Murphy, 2015) and is more consistent with the hypothesis that FLA4 acts as a soluble factor. This does not exclude a direct interaction of FLA4 with FEIs or other RLKs, but it suggests that, if such an interaction exists, it might be more consistent with classical ligand to receptor binding. Because constructs with relatively low extracellular abundance were genetically active, we regard it unlikely that FLA4 functions as a major structural component in the cell wall in the root. However, it might act as a quantitatively minor wall component (Tan *et al*., 2013) or as a hormone‐like glycoprotein. At present we cannot exclude either option and it is conceivable that similar to all four human fasciclin proteins, FLA4 might fulfil multiple cellular roles.

### FLA4 predominantly acts through its carboxy‐proximal Fas1 domain

FLA4 belongs to a large group of eukaryotic Fas1 proteins that contain one or more tandem repeats of the Fas1 domain. The functional significance of the tandem architecture remains controversial (Moody and Williamson, 2013). Our sequence comparison between putative *FLA4* orthologues and the FLA4 locus in different *Arabidopsis thaliana* accessions showed that the Fas1‐2 domain is more conserved than any other FLA4 region. This hints at a dominant functional role of the Fas1‐2 domain. Indeed, the deletion of the entire Fas1‐1 and PR1 domain demonstrated that the Fas1‐2 domain was sufficient for the function of *FLA4*. By contrast, *FLA4* function was disrupted to the level of the knock out allele *sos5‐2* both by the single aminoacid substitution in the Fas1‐2 domain in the *sos5‐1* allele or by deletion of a short sequence at the amino‐proximal region of the Fas1‐2 domain in the *F4C∆Fas1‐1∆PR1*.*6* construct. But how is this observation compatible with the evolutionary conservation of the tandem Fas1 architecture of FLA4 in orthologues? As an initial clue we observed that in growing roots the localization of the F4C∆Fas1‐1 protein in endosomes was dramatically increased compared with the mostly plasma membrane localized protein full‐length F4C. Because *N‐*glycosylation is involved in the apical sorting of mammalian GPI‐APs (Muniz and Riezman, 2016), one could speculate that the highly *N‐*glycosylated Fas1‐1 domain might be required for the efficient secretion of FLA4. However, several observations argue against this interpretation. For one there was a high presence of the fully functional *F4C∆Fas1‐1* constructs in the apoplast as indicated by profuse cell corner localization (Figure 5a) that was reminiscent of the localization of secreted reporter proteins (Teh and Moore, 2007). Secondly, ER‐exit as observed by confocal microscopy and *N‐*glycosidase digest was unaffected by the absence of the Fas1‐1 domain. This means that the Fas1‐1 domain appears to predominantly affect the post‐secretory fate of FLA4 where it stabilizes the protein at the plasma membrane. This behaviour is reminiscent of the fate of destabilized GPI‐APs in mammals and yeast that are not retained in the ER but are secreted and targeted to the lysosome for destruction. The molecular mechanism how abnormal GPI‐anchored proteins are preferentially directed to endosomes from the plasma membrane remains to be established (Satpute‐Krishnan *et al*., 2014; Sikorska *et al*., 2016).

### Protein glycosylation of either type is required for efficient protein transport and localization

We show that F4C is *N‐*glycosylated, mostly by α(1–3) core fucosylated complex *N‐*glycans and to a small extent with oligomannosidic glycans and that it carries AGP‐type *O‐*glycan epitopes. The most highly conserved *N‐*glycosylation site in the FLA family is at the amino‐proximal margin of the Fas1 domains and might be a key determinant for the subcellular trafficking within this group of proteins. Deletion of this glycosylation site in FLA4 led to predominant retention of the protein in the ER which is an example how an individual *N‐*glycan in multiple‐site glycosylated proteins can crucially affect protein localization. Individual *N‐*glycans can function in roles such as ligand binding, protein folding or ER‐exit. Similar to our results the substitution of two conserved *N‐*glycosylation sites N43 and N122 of the S‐locus receptor kinase (SRKb) led to ER‐retention without affecting genetic function (Yamamoto *et al*., 2014). The *N‐*glycan at N207 of FLA4 is positioned between a pair of hydrophobic amino acids (Figure S4) and the exposure of this group might lead to enhanced binding of ER chaperones and ER‐retention. Alternatively, the *N‐*glycan at this position might constitute a family‐wide ER‐exit signal that might determine differential localization for different FLA family members. We also present direct evidence that F4C contains AGP‐like glycans. F4C reacted with the two AGP‐glycan specific monoclonal antibodies LM14 (Moller *et al*., 2008) and JIM13 (Knox *et al*., 1991). In principle, cross reaction might also be explained by a co‐purifying AGP, however, also the mass‐shifted derivative F4C∆Fas1‐1, showed decoration with LM14 and JIM13. By contrast, F4C∆Fas1‐1∆PR1.6 that is trapped in the ER was not reactive with *O‐*glycan antibodies. Consistent with the prevailing view that the major part of an AGP‐glycan is synthesized in the Golgi, only the Endo‐H resistant F4C fraction that corresponded with Golgi‐processed FLA4 reacted with LM14 or JIM13. Likewise, the heterogeneous fraction of F4C (*mur1‐2*) that was released by PNGase F treatment and that appeared 7–17 kDa larger than the 66 kDa fragment, corresponded to the *O‐*glycosylated and de‐*N‐*glycosylated fraction of F4C. The relatively homogeneous molecular weight of F4C around 91 kDa suggests that mature native FLA4 might have a molecular weight of around 62 kDa including *N‐* and *O‐*glycosylation. This number is in good agreement with data for poplar PtFLA6 (Wang *et al*., 2015), and also matches well with purified FLA2 that migrated as a 66 kDa glycoprotein (Murphy *et al*., 2002). We addressed the question to which aminoacid residues of FLA4 AG‐chains might be attached by replacing all conserved clustered proline residues by alanine. The mutant protein showed increased fragmentation and a reduced reactivity with the JIM13 probe but still reacted with the LM14 antibody. This suggests AGs that carry the JIM13 epitope might be attached to clustered Pro residues while LM14 epitope carrying glycans might be linked to residues yet to be identified. The clustered proline residues in the PR domains are redundant for FLA4 function. As especially the proline clusters in the PR2 domain are conserved, we suggest that they might play a role that is not phenotypically apparent under the used conditions. The proline clusters on FLA4 clearly are important for protein localization and abundance at the plasma membrane. As our manipulations failed to fully suppress *O‐*glycosylation, additional studies will be required to test the hypothesis that *O‐*glycans might be instrumental for receptor recognition as previously hypothesized (Basu *et al*., 2016; Showalter and Basu, 2016), however, here we suggest a crucial role for the *O‐*glycan modifications for the cellular fate of FLA4, as was previously suggested for a chimeric AGP promoting somatic embryogenesis in cotton cultures (Poon *et al*., 2012). By generating a functional reporter for FLA4 we provided a paradigm for the localization, trafficking and cellular processing of FLAs. By extrapolation our work suggests that the Fas1‐2 domains plays the crucial role in the action in this group of extracellular proteins. Structural motifs such as tandem Fas1 organization, GPI‐anchoring and glycosylation appear to support the Fas1‐2 domain in reaching its site of action and to influence its biological stability. Apart from the identification of molecular interactors, an important future challenge is the precise definition of *O‐*glycosylation sites and *O‐*glycan structure to better understand the implication of this type of modification on protein trafficking and function.

## Experimental procedures

### Growth conditions and measurements

Wild‐type *Arabidopsis thaliana* seeds of the ecotype Col gl and *sos5‐1* seeds and homozygous sos5‐2 seeds were kindly provided by Jian‐Kang Zhu (Univ. California, Riverside) and Joseph Kieber (Univ. North Carolina, Chapel Hill), respectively. Growth conditions and root measurement and salt treatment were as previously described (Seifert *et al*., 2014).

### Plasmid constructs

The FLA4:F4C construct was based on pGREEN179 (GenBank: EU048866.1) (Hellens *et al*., 2000) plant transformation vector and contains PCR fragments of the 1000‐nt promoter region including the 5′UTR, the 27 residue secretion signal of FLA4 (residues 1–27), a SRVPV linker, mCitrin (Shaner *et al*., 2005) (GenBank: AEJ82308.1; residues 1–239), followed by most of the FLA4 coding region (At3g46550: residues 29–420) and the HSP18.2 (At5g59720) terminator (Nagaya *et al*., 2009). The 635‐nt promoter region of the UBQ10 locus was used for the indicated constructs. All oligonucleotide combinations are shown in table S2. The Pro to Ala substitution F4C_P1234A contains an artificial gene (Genscript Ltd, Hong Kong) where the all codons corresponding to clustered Pro residues P192 to P194, P201 to P203, P368/P369 and P389/P390 were replaced with Ala codons. The N207Q substitution was introduced by overlap extension PCR (Bryksin and Matsumura, 2010). After transformation of the constructs into *Agrobacterium* strain GV3101 (pre‐transformed with pSOUP) we transformed *sos5‐1* plants by floral dip (Clough and Bent, 1998). The transformed T1 seedlings are selected on standard medium containing 40 mg/L hygromycin. For selected lines, the T‐DNA insertion allele *sos5‐2* (Col‐0) was crossed with transformant lines to generate a homozygous *sos5‐2* ^*−/−*^ (*Col‐0/Col gl*) background to assess the potential genetic interactions of the endogenous *sos5‐1* mutant protein with the recombinant FLA4‐citrin (F4C) protein or its variants.

### Electrophoresis and western blotting

Tissues were frozen in liquid nitrogen and ground to powder in a frozen state in a ball mill (Retsch, Germany) for 2 min at 30 min^−1^. Protein was extracted using 50 mm Tris–HCl pH 8.0, 20 mm EDTA, 1 mm DTT and 1% proteinase inhibitors cocktail (Sigma, P9599). Before electrophoresis the samples were heated at 95°C for 5 min in the presence of loading buffer (50 mm Tris–HCl pH 6.8, 2% SDS, 10% glycerol, 100 mm DTT, 12.5 mm EDTA, 0.02% bromophenol blue). The proteins were separated on an 8.5% SDS‐PAGE according to (Laemmli, 1970) and electro‐blotted onto nitrocellulose membrane (Amersham Protran, GE Healthcare Life Science) at 100 V for 45 min. The membrane was blocked with 2.5% skimmed dried milk (in PBST buffer) for 1 h. The citrin tag was detected with anti‐GFP (D5.1) XP^®^ Rabbit mAb (Cell Signalling; 1:1000 in blocking buffer) followed by horseradish peroxidase‐conjugated anti‐rabbit secondary antibody (Cell signalling; 1:10 000 in PBST) and Amersham ECL Prime Western Blotting Detection Reagent (Amersham, GE Healthcare Life science). Blots were exposed to X‐ray film (Amersham, GE Healthcare Life Science) and recorded on a ChemiDoc XRS Imager (Bio‐Rad). Endo H (New England BioLabs) and PNGase F (New England BioLabs) digestions were performed as previously described (Hüttner *et al*., 2012).

### Microsome preparation and GPI‐PLC digest

Snap frozen tissue was homogenized using a ball mill and protein was extracted using 50 mm Tris–HCl pH 8.0, 400 mm sucrose, 20 mm EDTA, 1 mm DTT and 1% proteinase inhibitor cocktail (Sigma, P9599). The insoluble debris was removed by centrifugation at 300 ***g*** for 10 min at 4°C. The 300 g cell wall pellet is washed once. Microsomal membranes were separated from soluble proteins by centrifugation at 100 000 ***g*** for 1 h at 4°C. Two‐phase partitioning using Triton X‐114 and GPI‐PLC digest was performed as previously described (Sherrier *et al*., 1999).

### Affinity purification of citrin tagged FLA4 derivatives

We used the μMACS and MultiMACS GFP Isolation Kits (Miltenyi Biotec) to purify citrin tagged protein according to manufacturer's instruction with the following modifications. Binding of 400 μl extract to 50 μl anti‐GFP‐tagged magnetic beads was done at 4°C for 20 h. Washing was done with 6 × 200 μl of extraction buffer including 0.1% SDS and a final rinse with 100 μl wash buffer (50 mm Tris–HCl, pH 8.0) in a μMACS magnetic separator. We eluted the protein from the column with 80 μl of SDS‐PAGE loading buffer (Laemmli, 1970) preheated to 95°C. Gels were stained with SYPRO Ruby (Thermo Fisher Sci.) for subsequent protein identification using LC‐ESI‐MS/MS.

### Protein identification and peptide analysis using LC‐ESI‐MS/MS

We excised relevant protein bands and performed in gel digest ion by S‐alkylation with iodoacetamide and digestion with sequencing grade modified trypsin (Promega). We analysed the peptide mixture as described (Pabst *et al*., 2012) using a Dionex Ultimate 3000 system directly linked to quadrupole ion trap instrument (amaZon speed ETD, Bruker) equipped with the standard ESI source in the positive ion, DDA mode. We converted analysis files to XML files, using Data Analysis 4.0 (Bruker) which subjects MS/MS ion searches to MASCOT (embedded in ProteinScape 3.0, Bruker) for protein identification. Only proteins identified with at least two peptides with a protein score higher than 80 were accepted. For the searches the SwissProt database was used.

### Detection of cell wall carbohydrate epitopes and *O‐*glycosidase digest

After membrane transfer, the purified protein was probed with monoclonal rat antibodies specific for various cell wall carbohydrate epitopes. For these antibodies, membranes were blocked with 3% BSA in PBST for 1 h. The antibodies were used at a dilution of 1:50 blocking buffer and were detected with anti‐Rat IgM horseradish peroxidase antibody (Jackson ImmunoResearch Laboratories) (1:10 000) followed by chemiluminescence detection (Amersham, GE Healthcare Life Science). In this study the AGP antibodies LM14 (Moller *et al*., 2008), JIM13 (Knox *et al*., 1991), LM2 (Smallwood *et al*., 1996), JIM8 (Pennell *et al*., 1991), JIM16(Yates *et al*., 1996), and KM1 (Classen *et al*., 2004) as well as the pectin associated epitopes recognised by the monoclonal antibodies JIM5 (Clausen *et al*., 2003) and LM6 (Willats *et al*., 1998) and the extensin related antibodies LM3, JIM11, JIM12, JIM20 (Smallwood *et al*., 1996) and JIM19 (Knox *et al*., 1995) were tested. Affinity‐purified F4C was digested with the AG‐specific enzymes α‐l‐arabinofuranosidase, β‐glucuronidase and exo‐β‐(1→3)‐galactanase as described (Tryfona *et al*., 2012).

### Confocal microscopy

We mounted seedlings in dH_2_O for general observation or in 0.5 m mannitol for plasmolysis experiments immediately before observation. BFA was used at 20 μm in dH_2_O and For confocal laser scanning microscopy we use a Leica SP5 or SP8 STED both equipped with HyD detectors and *a* × 63/1.2 water immersion lens. STED was performed with *a* × 100/1.4 oil immersion lens. Images were imported and processed with FIJI (ImageJ) freeware and mounted with Adobe Photoshop. Unless otherwise indicated imaging settings and contrast operations were identical between compared specimens.

### Bioinformatics

Putative FLA4 orthologue were identified in the plant genome duplication database (PGDD; http://chibba.agtec.uga.edu/duplication/). Before sequence alignment, using Clustal Omega (http://www.ebi.ac.uk/Tools/msa/clustalo/) (Sievers *et al*., 2011), *N‐*terminal secretion and C‐terminal GPI‐modification signal sequences were predicted using SignalP 4.1 (http://www.cbs.dtu.dk/services/SignalP/) (Petersen *et al*., 2011) and BIG‐PI Plant predictor (http://mendel.imp.ac.at/gpi/plant_server.html) (Eisenhaber *et al*., 2003) and removed. Non‐synonymous sequence polymorphisms between *Arabidopsis thaliana* accessions were identified in the 1001 genomes database (Consortium TG, 2016) using the 1001 Proteomes tool.

### Accession numbers

IDs of aligned sequences: tomato ref|XP_004236212.1|, riceFLA4A ref|XP_015638227.1|, MusaFLA4X2 ref|XP_009402266.1|, MusaFLA4 ref|XP_009384845.1|, Malus domestica FLA4 ref|XP_008385122.1|, EucalyptFLA4 ref|XP_010043558.1|.

## Conflict of Interest

The authors declare no conflict of interest.

## Supporting information


**Figure S1**. FLA4‐citrin domains and key features.

**Figure S2**. Treatment with GPI‐PLC removes F4C and SKU5 from the membrane fraction.

**Figure S3**. The C‐terminal putative GPI‐modification signal sequence is not required for genetic function of F4C.

**Figure S4**. Alignment of putative FLA4 orthologues.

**Figure S5**. Peptide sequence polymorphisms in AtFLA4 in various Arabidopsis accessions.

**Figure S6**. *N‐*proximal Fas1‐1 region and the PR1 domain are not required for complementing *sos5‐1*.

**Figure S7**. Brefeldin A leads to redistribution of F4C and F4C∆Fas1‐1 into BFA bodies.

**Figure S8**. Localization of several constructs that lack the Fas1‐1 domain and to different extent the PR1 domain.

**Figure S9**. Positioning of predicted *N‐*glycosylation sites in Fas1 domains of *Arabidopsis thaliana* FLAs.

**Figure S10**. The *N‐*glycosylation site N207 is not required for F4C function in root growth and NaCl tolerance.

**Figure S11**. Detailed protein report on peptides identified in the two excised protein bands visible after F4C immuno‐affinity purification.

**Figure S12.** Inhibitor of prolyl4‐hydroxylase bipyridyl (BP) suppresses F4C abundance.

**Figure S13**. The clustered proline residues in the two PR domains are not required for FLA4 function in root growth and NaCl tolerance.

**Table S1.** Conserved *N‐*glycosylation sites at *N‐*terminal margin of Fas1 domains in Arabidopsis FLAs.

**Table S2.** Oligonucleotide primers used in this study.
