# Supplementary material for: Arabidopsis thaliana FLA4 functions as a glycan‐stabilized soluble factor via its carboxy‐proximal Fasciclin 1 domain
Source: Plant J. 2017 Jun 13;91(4):613–30. doi: 10.1111/tpj.13591 (PMC5575511; doi:10.1111/tpj.13591)
Supplement: Supplementary file 1 — Figure S1. FLA4‐citrin domains and key features. [file TPJ-91-613-s001.pdf]

MANVISISHFTLLALPYLLLLLSSTAAASRVPVMVSKGEELFTGVVPILVELDG
DVNGHKFSVSGEGEGDATYGKLTLKFICTTGKLPVPWPTLVTTFGYGLMCFA
RYPDHMKQHDFFKSAMPEGYVQERTIFFKDDGNYKTRAEVKFEGDTLVNRI
ELKGIDFKEDGNILGHKLEYNYNSHNVYIMADKQKNGIKVNFKIRHNIEDGSV
QLADHYQQNTPIGDGPVLLPDNHYLSYQSALSKDPNEKRDHMVLLEFVTAA
GITLGMDELYKINVTAVLSSFPNLSSFSNLLVSSGIAAELSGRNSLTLLAVPNSQFSS
ASLDLTRRLPPSALADLLRFHVLLQFLSDSDLRRIPPSGSAVTTLYEASGRTFFGSG
SVNVTRDPASGSVTIGSPATKNVTVLKLLETKPPNITVLTVDSLIVPTGIDITASETLTP
PPTSTSLSPPPAGINLTQILINGHNFNVALSLLVASGVITEFENDERGAGITVFVP
TDSAFSDLPSNVNLQSLPAEQKAFVLKFHVLHSYYTLGSLESITNPVQPTLAT
EEMGAGSYTLNISRVNGSIVTINSGVVLAVVTQTAFDQNPVSVFGVSKVLLPK
ELFPKSGQPVATAPPQEISLSPESSSEQPSRLVSPPREIVSSGAVKRPLGFLV
LWCWCIAFCYVLV

supplemental Figure S1: FLA4-citrin sequence, domains and key features. N-terminal signal peptide predicted by SignalP4.1 (underlined). mCitrin (yellow), N-proximal Fas1-1 domain (dark green), C-proximal Fas1-2 domain (light green), H-regions (pink), N-glycosylation sites (NXS/T; purple), Ser mutated to Phe in *sos5-1* (red) clustered Pro-residues in Pro-rich regions (olive), GPI-modification signal (weakly) predicted by BIG-PI Plant predictor (grey) are highlighted.
