# Supplementary figures and images for: Arabidopsis thaliana FLA4 functions as a glycan‐stabilized soluble factor via its carboxy‐proximal Fasciclin 1 domain
Source: Plant J. 2017 Jun 13;91(4):613–30. doi: 10.1111/tpj.13591 (PMC5575511; doi:10.1111/tpj.13591)

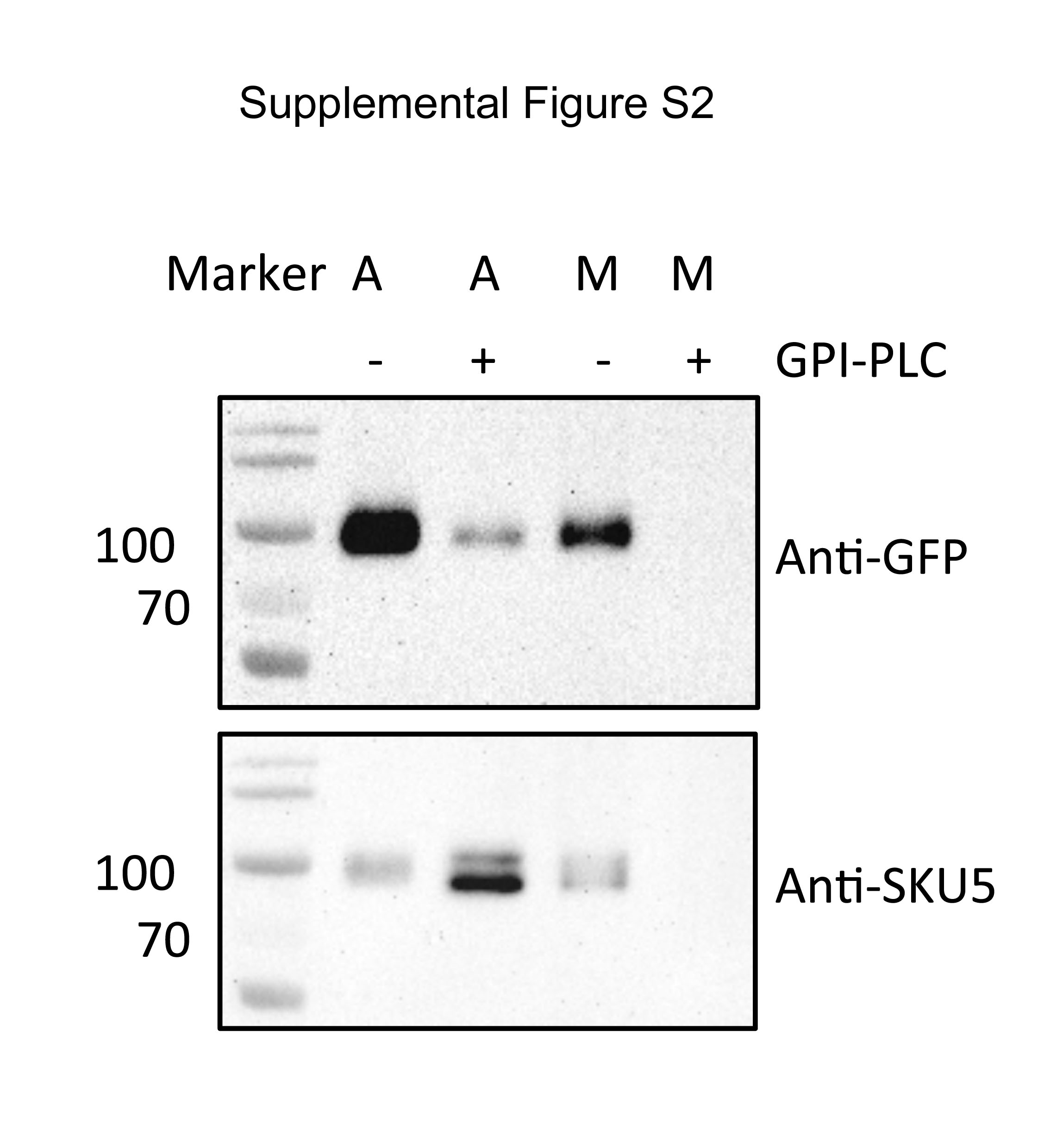

Supplement: Supplementary file 2 — Figure S2. Treatment with GPI‐PLC removes F4C and SKU5 from the membrane fraction. [file TPJ-91-613-s002.jpg]

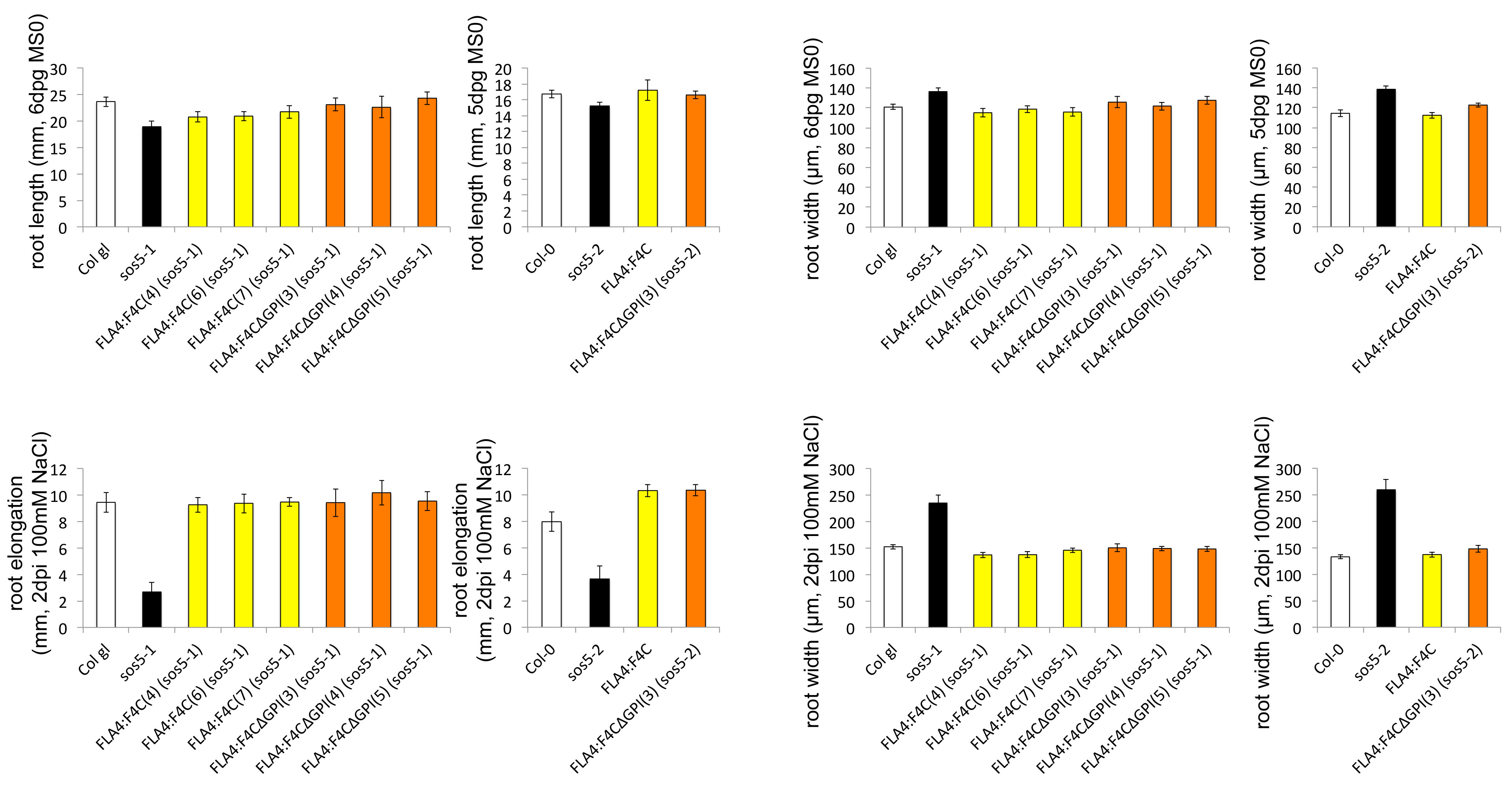

Supplement: Supplementary file 3 — Figure S3. The C‐terminal putative GPI‐modification signal sequence is not required for genetic function of F4C. [file TPJ-91-613-s003.jpg]

supplemental Figure S4

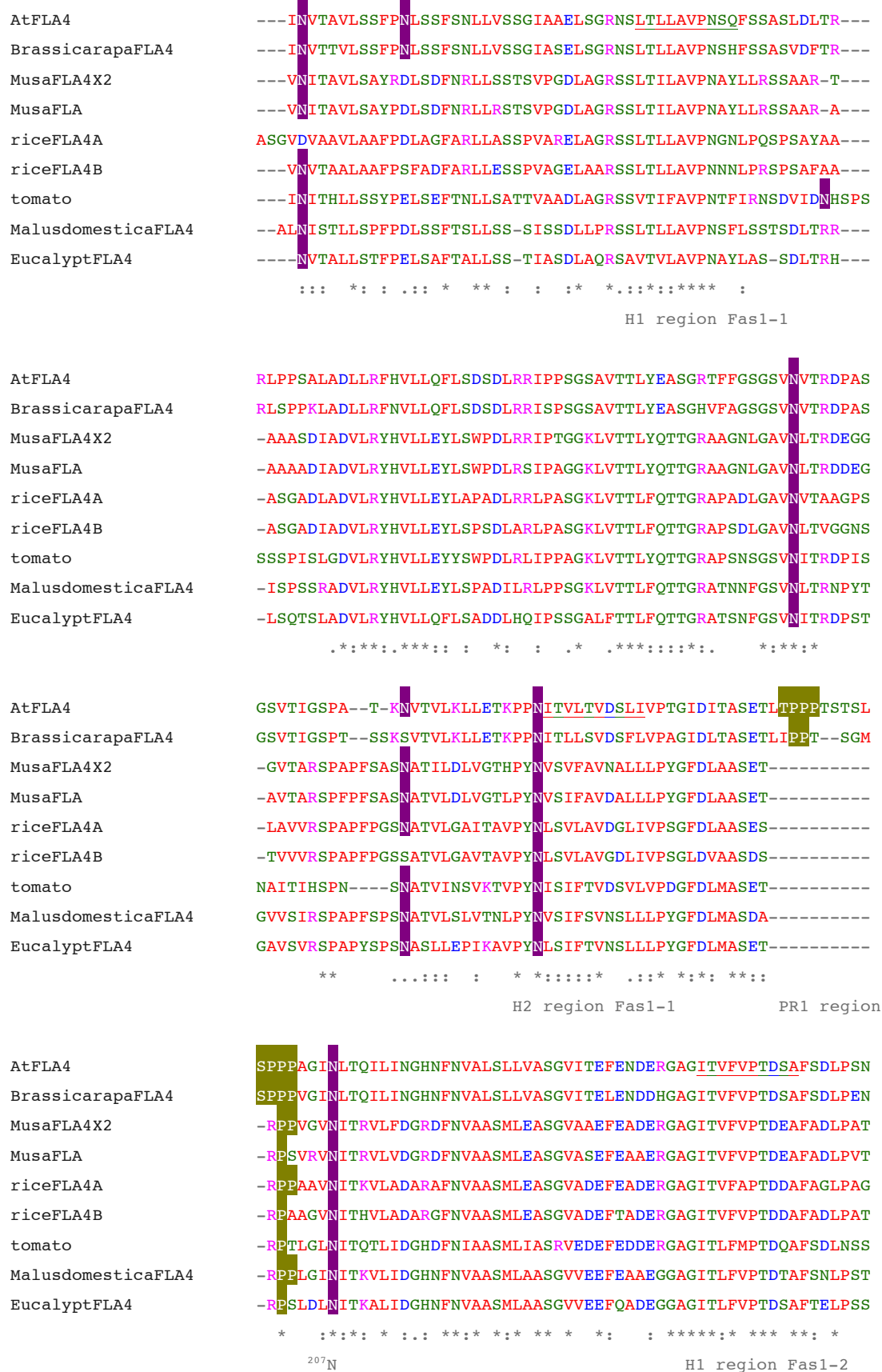

Supplement: Supplementary file 4 — Figure S4. Alignment of putative FLA4 orthologues. [file TPJ-91-613-s004.pdf]

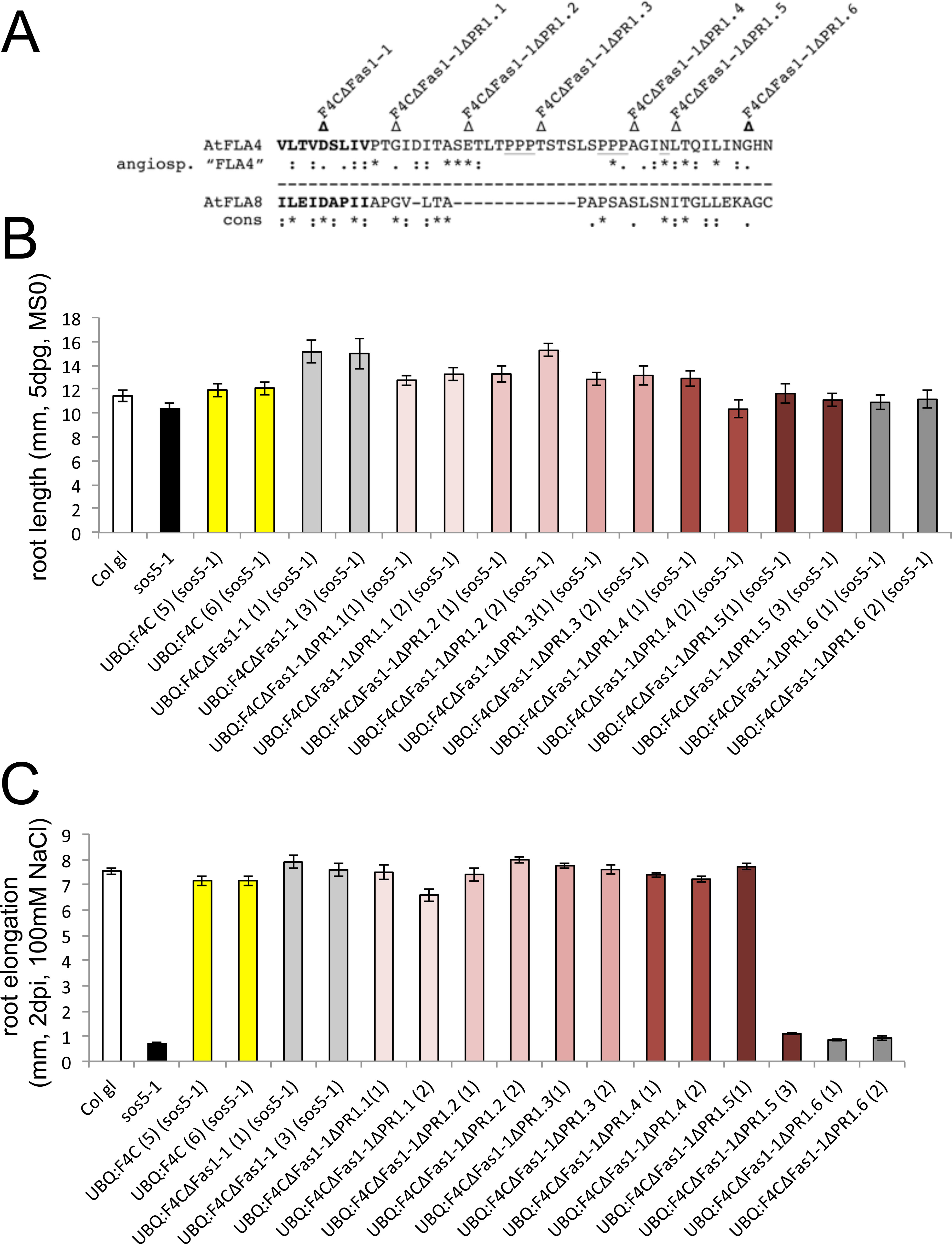

Supplement: Supplementary file 6 — Figure S6. N‐proximal Fas1‐1 region and the PR1 domain are not required for complementing sos5‐1. [file TPJ-91-613-s006.jpg]

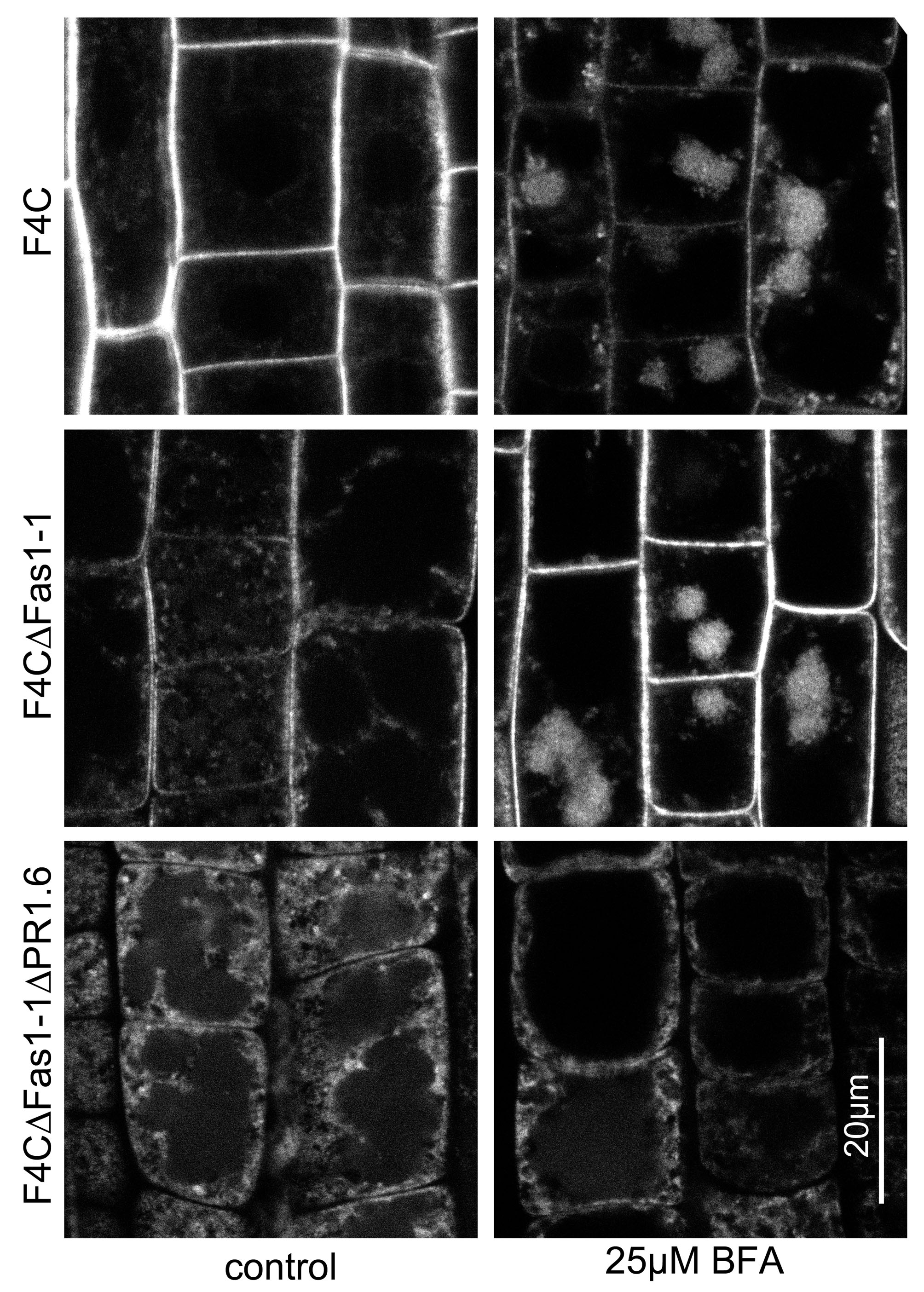

Supplement: Supplementary file 7 — Figure S7. Brefeldin A leads to redistribution of F4C and F4C∆Fas1‐1 into BFA bodies. [file TPJ-91-613-s007.jpg]

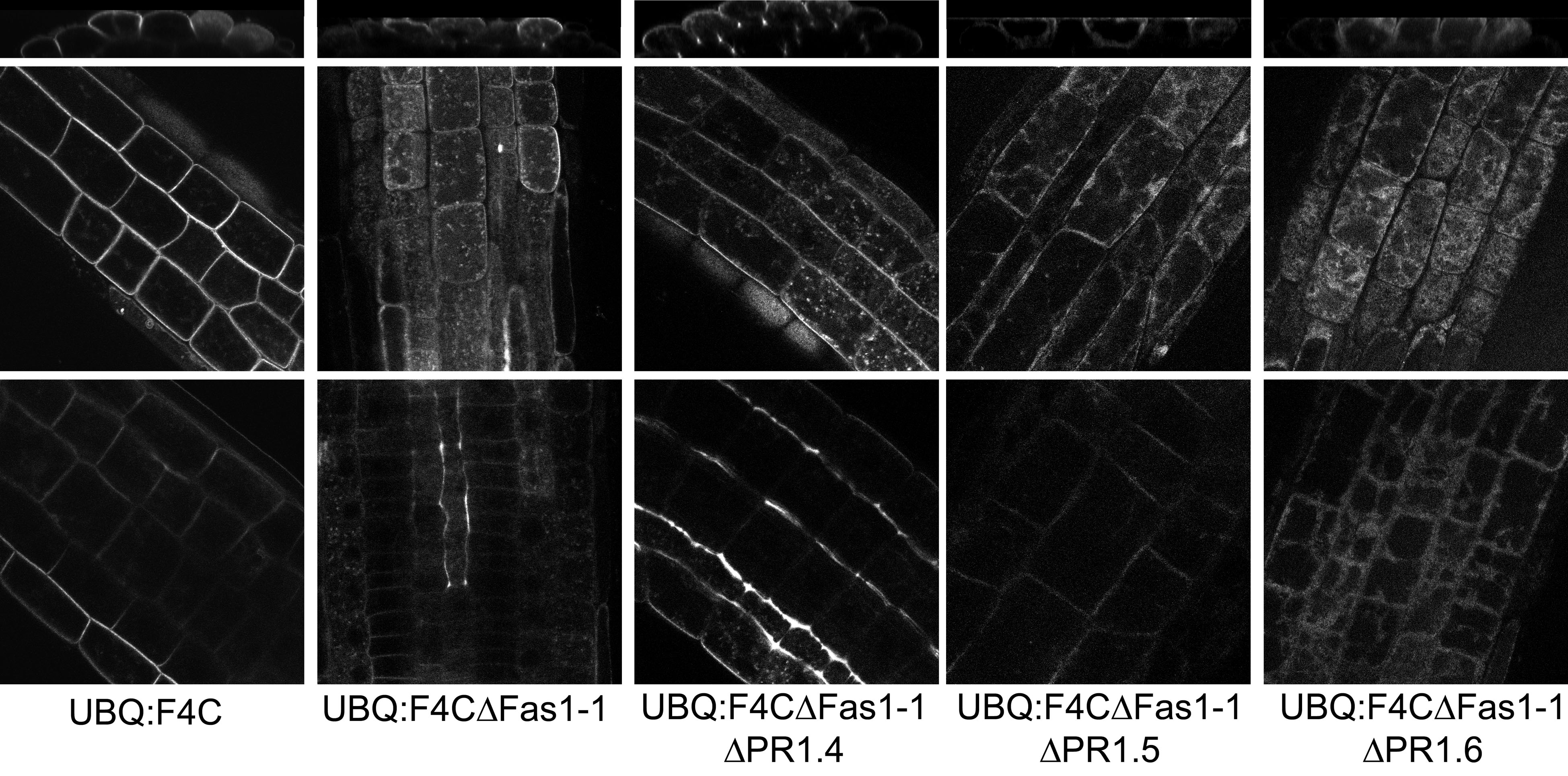

Supplement: Supplementary file 8 — Figure S8. Localization of several constructs that lack the Fas1‐1 domain and to different extent the PR1 domain. [file TPJ-91-613-s008.jpg]

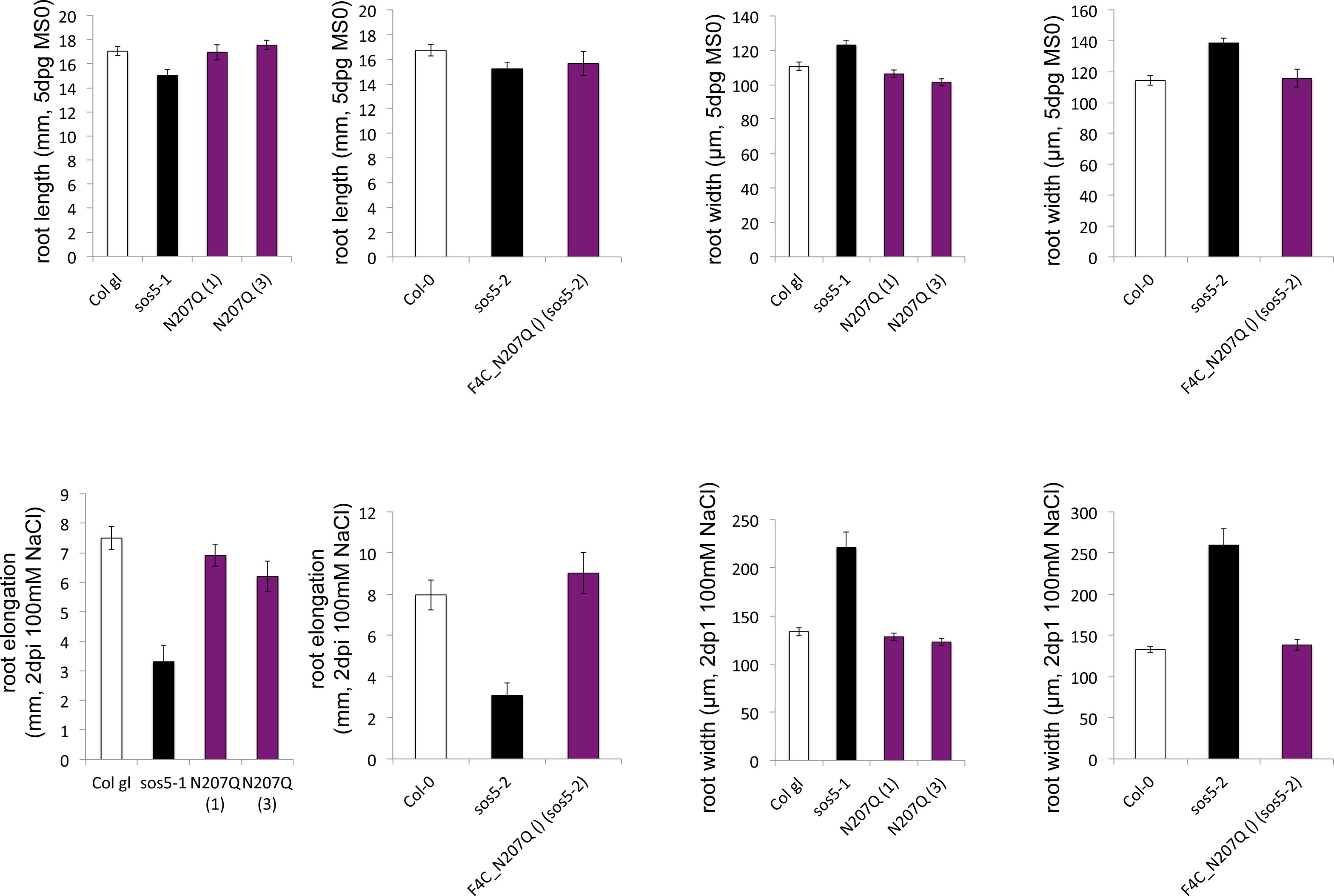

Supplement: Supplementary file 10 — Figure S10. The N‐glycosylation site N207 is not required for F4C function in root growth and NaCl tolerance. [file TPJ-91-613-s010.jpg]

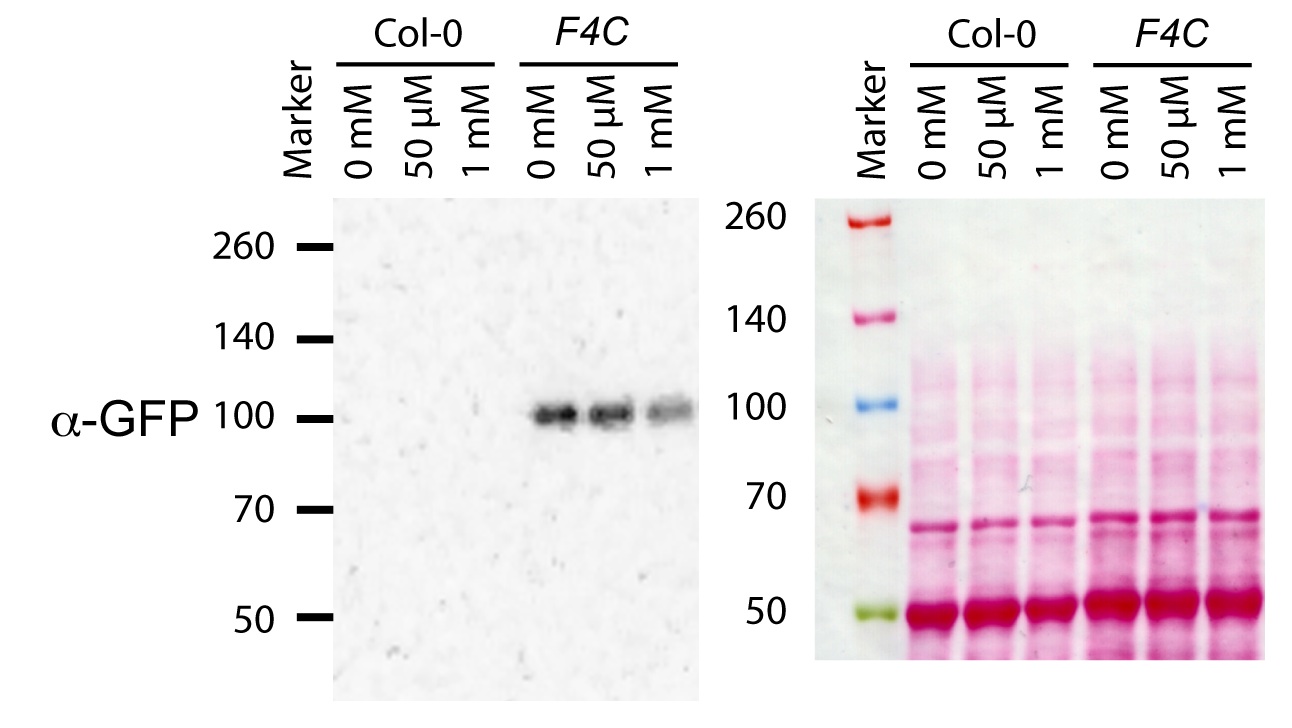

Supplement: Supplementary file 12 — Figure S12. Inhibitor of prolyl4‐hydroxylase bipyridyl (BP) suppresses F4C abundance. [file TPJ-91-613-s012.jpg]

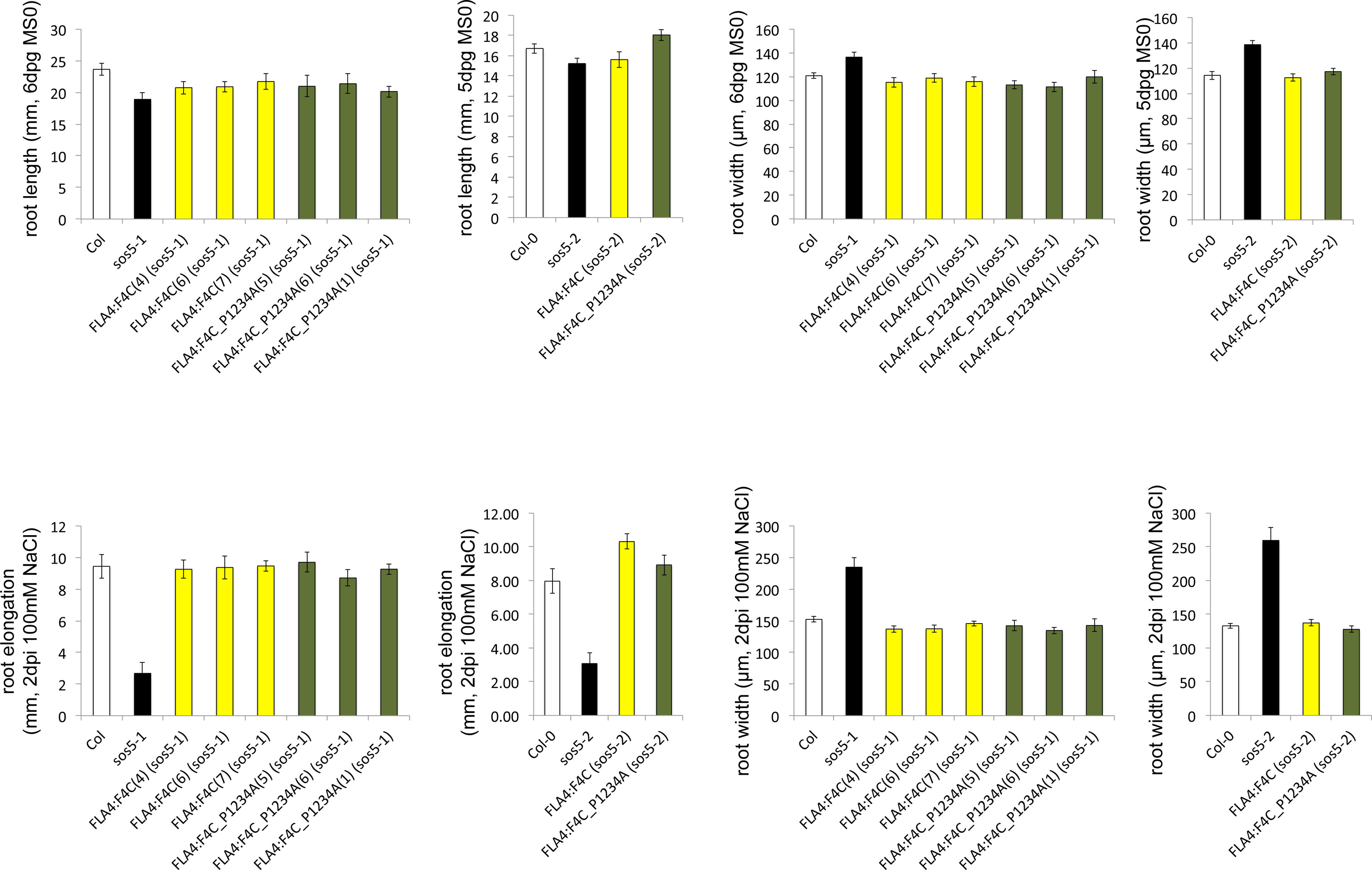

Supplement: Supplementary file 13 — Figure S13. The clustered proline residues in the two PR domains are not required for FLA4 function in root growth and NaCl tolerance. [file TPJ-91-613-s013.jpg]
