# Supplementary material for: Arabidopsis thaliana FLA4 functions as a glycan‐stabilized soluble factor via its carboxy‐proximal Fasciclin 1 domain
Source: Plant J. 2017 Jun 13;91(4):613–30. doi: 10.1111/tpj.13591 (PMC5575511; doi:10.1111/tpj.13591)
Supplement: Supplementary file 5 — Figure S5. Peptide sequence polymorphisms in AtFLA4 in various Arabidopsis accessions. [file TPJ-91-613-s005.pdf]

supplemental Figure S5

```
MANVISISHFTLLALPYLLLLLLSSTAAAINVTAVLSSFPNLSSFSNLLVSSGIAAELSGRNSLTLLA
            C              A                V                 T  S S                      V
                                                                   Y                      T

VPNSQFSSASLDLTRRLPPSALADLLRFHVLLQFLSDSDLRRIPPSGSAVTTLYEASGRTFFGSGS
P            D            C                                                    A

VNVTRDPASGSVTIGSPATKNVTVLKLLETKPPNITVLTVDSLIVPTGIDITASETLTPPPTSTSL
          A    L                  M      D                                     M

SPPPAGINLTQILINGHNFNVALSLLVASGVITEFENDERGAGITVFVPTDSAFSDLPSNVNLQSL
P

PAEQKAFVLKFHVLHSYYTLGSLESITNPVQPTLATEEMGAGSYTLNISRVNGSIVTINSGVVLA
                       A

VVTQTAFDQNPVSVFGVSKVLLPKELFPKSGQPVATAPPQEISLSPESSSEQPSRLVSPPREIVSS
          V                         I                 I            L     F      N G

GAVKRPLGFLVLWCWCIAFCYVLV
C    E   T        G              LY
S
```

section	length	divergent	conserved (.)	highly conserved (:)
NSP	28	1	1	1
Fas1-1	151	8	2	1
PR1	27	2	-	-
Fas1-2	145	-	-	2
PR2	44	3	2	1
GPI	25	5	1	-

supplemental Figure S5: Peptide sequence polymorphisms in AtFLA4 in various Arabidopsis accessions revealed by the 1001 genomes project. The Col-0 sequence is boxed green in the two Fas1 domains and pink in the H1 and H2 domains. Predicted N-glycosylation sites are boxed purple. Clustered proline residue in PR regions are boxed olive. Polymorphic residues are boxed yellow with the substituted residues shown underneath. The table below summarizes the occurrence of non-synonymous polymorphisms in the various functional domains of AtFLA4 by degree of conservation according to CLUSTAL. Note the relatively high degree of conservation in the entire Fas1-2 domain.
