# Supplementary material for: Arabidopsis thaliana FLA4 functions as a glycan‐stabilized soluble factor via its carboxy‐proximal Fasciclin 1 domain
Source: Plant J. 2017 Jun 13;91(4):613–30. doi: 10.1111/tpj.13591 (PMC5575511; doi:10.1111/tpj.13591)
Supplement: Supplementary file 9 — Figure S9. Positioning of predicted N‐glycosylation sites in Fas1 domains of Arabidopsis thaliana FLAs. [file TPJ-91-613-s009.pdf]

supplemental Figure S9

```
FLA15Fas1-1      ----------------------NSNSVLVALLDSHYTELAELVE-KALLLQTLEEAVG--
Fas16Fas1-1      ----------------------NSNSVLVALLDSHYTELAELVE-KALLLQTLEEAVG--
FLA17Fas1-1      ----------------------NSNSVLVALLDSRYTELAELVE-KALLLQTLEDAVG--
FLA18Fas1-1      FFLLNASALESGHHNITGSGQINSNSVLVALLDSRYTELAELVE-KALLLQTLEDAVG--
FLA15Fas1-2      ----------AQVKDFIH----------TLLHYGGYNEMADILVNLTSLATEMGRL-VS-
Fas16Fas1-2      ----------AQVKDFIH----------TLLHYGGYNEMADILVNLTSLATEMGRL-VS-
FLA17Fas1-2      ----------AQVKDFIH----------TLLHYGGYNEMADILVNLTSLATEMGRL-VS-
FLA18Fas1-2      ----------AQVKDFIH----------TLLHYGGYNEMADILVNLTSLATEMGRL-VS-
FLA7             -------APAPENVNLTE----------LLSVAGPFHTFLDYLL-STGVIETFQNQANN-
FLA6             --------TEKSPINLTA----------ILEAGHQFTTLIQLLN-TTQVGFQVSVQLNS-
FLA9             ----------AGPINLTA----------ILEKGGQFTTFIHLLN-ITQVGSQVNIQVNS-
FLA13            ---------PAGPINITA----------ILEKGGQFVTLIRLLN-TTQIGNQINIQINS-
FLA11            ---------PSGPTNITA----------ILEKAGQFTLFIRLLK-STQASDQINTQLNSS
FLA12            ----------PGPTNVTK----------ILEKAGQFTVFIRLLK-STGVANQLYGQLNN-
FLA4Fas1-2       ---------PPAGINLTQ----------ILINGHNFNVALSLLV-ASGVITEFEND---E
FLA8Fas1-2       -------APSASLSNITG----------LLEKAGCKTFANL-LV-SSGVLKTYESA----
FLA10Fas1-2      ---------SAGVSNITG----------LLEKAGCKTFANL-LV-SSGVIKTFEST----
FLA1Fas1-2       ------------EMNLTG----------IMSAHGCKVFAETLLT-NPGASKTYQES----
FLA2Fas1-2       ---------SPSDLILTT----------ILEKQGCKAFSDI-LK-STGADKTFQDT----
FLA4Fas1-1       -------------INVTA----------VLSSFPNLSSFSNLLV-SSGIAAELSGR----
FLA3             -------SSIVSAVNITR----------VLEKYPEFSTMTELLA-KTELTPIINKR----
FLA5             ------FSKVVTANNITL----------AFQKYSKFSTMRDLFI-KTKLIAAIDKY----
FLA14            ----------SNSFNITN----------ILNEHDDFSNFNQLLS-ETQLASTINKR----
FLA8Fas1-1       -------------HNITQ----------ILADSPDYSSFNSYLS-QTKLADEINSR----
FLA10Fas1-1      -----------SGHNITQ----------ILSDTPEYSSFNNYLS-QTKLADEINSR----
FLA1Fas1-1       --------------NVTR----------LLANHPSFSSFSHFLT-QTHLADEINRR----
FLA2Fas1-1       --------------NITR----------ILAKDPDFSTFNHYLS-ATHLADEINRR----
                                                           .
                               N30                 N40
                               N207
```

supplemental Figure S9 (contd.)

```
FLA15Fas1-1      -QHNITIFAPRNDALEKNL-DPEFKSFLLQPKNLKSLQSLLMFHILPKRITSPQFSSAV-
Fas16Fas1-1      -KHNITIFAPRNDALERNL-DPLFKSFLLEPRNLKSLQSLLMFHILPKRITSPQWPSLS-
FLA17Fas1-1      -RHNITIFAPRNEALERDL-DPEFKRFLLEPGNLKSLQTLLMFHIIPNRVGSNQWPSEE-
FLA18Fas1-1      -RHNITIFAPRNEALERDL-DPDFKRFLLQPGNLKSLQTLLLSHIIPKRVGSNQWPEEN-
FLA15Fas1-2      EGYVLTVLAPNDEAMAKLT-TDQLS-------EPGAPEQIMYYHIIPEYQTEESMYNSVR
Fas16Fas1-2      EGYVLTVLAPNDEAMAKLT-TDQLS-------EPGAPEQIMYYHIIPEYQTEESMYNAVR
FLA17Fas1-2      EGYVLTVLAPNDEAMAKLT-TDQLS-------EPGAPEQIVYYHIIPEYQTEESMYNSVR
FLA18Fas1-2      EGYVLTVLAPNDEAMGKLT-TDQLS-------EPGAPEQIMYYHIIPEYQTEESMYNSVR
FLA7             TEEGITIFVPKDDAFKAQK-NPPLSNL-----TKDQLKQLVLFHALPHYYSLSEFKNLS-
FLA6             SDQGMTIFAPTDNAFNKLK-PGTLNSL-----TYQQQIQLMLYHIIPKYYSLSDLLLAS-
FLA9             SSEGMTVFAPTDNAFQNLK-PGTLNQL-----SPDDQVKLILYHVSPKYYSMDDLLSVS-
FLA13            SSEGMTVLAPTDNAFQNLK-PGTLNKL-----SPDDQVKLILYHVSPKFYTLEDLLSVS-
FLA11            SSNGLTVFAPTDNAFNSLK-SGTLNSL-----SDQQKVQLVQFHVLPTLITMPQFQTVS-
FLA12            SDNGITIFAPSDSSFTGLK-AGTLNSL-----TDEQQVELIQFHVIPSYVSSSNFQTIS-
FLA4Fas1-2       RGAGITVFVPTDSAFSDLPSNVNLQSL-----PAEQKAFVLKFHVLHSYYTLGSLESIT-
FLA8Fas1-2       VEKGLTVFAPSDEAFKAEG-VPDLTKL-----TQAEVVSLLEYHALAEYKPKGSLKTNK-
FLA10Fas1-2      VEKGLTVFAPSDEAFKARG-VPDLTNL-----TQAEVVSLLEYHALAEYKPKGSLKTNK-
FLA1Fas1-2       LEGGMTVFCPGDDAMKG-F-LPKYKNL-----TAPKKEAFLDFLAVPTYYSMAMLKSNN-
FLA2Fas1-2       VDGGLTVFCPSDSAVGK-F-MPKFKSL-----SPANKTALVLYHGMPVYQSLQMLRSGN-
FLA4Fas1-1       --NSLTLLAVPNSQFSSAS-LDLTRRL-----PPSALADLLRFHVLLQFLSDSDLRRIP-
FLA3             --QTITVLALNNDAIGSIS-G-----R-----PEEEVKNILMNHVVLDYFDELKLKALK-
FLA5             --QTITVLAVSNDAISSIT-N-----R-----SEVELRNILMTHVILDYYDELKLQGMR-
FLA14            --QTITVLVVSNGALSSLS-G-----Q-----PTSVIKKILSLHIVLDYYDQKKLKNLS-
FLA8Fas1-1       --TTITVLVLNNGAMSALA-G---K-H-----PLSVIKSALSLLVLLDYYDPQKLHKIS-
FLA10Fas1-1      --TTITVLVLNNGAMSSLA-G---K-H-----PLSVVKNALSLLVLLDYYDPLKLHQLS-
FLA1Fas1-1       --RTITVCAVDNAAMSALT-S---KGY-----TLSTLKNILSLHVLLDYFGTKKLHQIR-
FLA2Fas1-1       --QTITVLAVDNSAMSSIL-S---NGY-----SLYQIRNILSLHVLVDYFGTKKLHQIT-
                     :*:    :  .                         :
                     H1 region
```

supplemental Figure S9 (contd.)

```
FLA15Fas1-1      ---VSHRT--LSND---HLH----FTN------GKVNSA--------EITKPDDLTRPDG
Fas16Fas1-1      ---HHHRT--LSND---HLH----LTVD--VNTLKVDSA--------EIIRPDDVIRPDG
FLA17Fas1-1      SGRVKHHT--LGND---QVR----LSNG--QGKKMVDLA--------EIIRPDDLTRPDG
FLA18Fas1-1      SGRVKHVT--LGHD---QVLHLSKLKGT--NGKRLVNSA--------VITRPDDLTRPDG
FLA15Fas1-2      RFGKIR---Y------DSLRFPHKVEAQEADGSVKFGHGDGSAY----LFDPDIYTDGRI
Fas16Fas1-2      RFGKVK---Y------DSLRFPHKVLAQEADGSVKFGHGDGSAY----LFDPDIYTDGRI
FLA17Fas1-2      RFGKVK---F------DTLRFPHKVAAKEADGSVKFGDGEKSAY----LFDPDIYTDGRI
FLA18Fas1-2      RFGKVK---Y------ETLRFPHKVGAKEADGSVKFGSGDRSAY----LFDPDIYTDGRI
FLA7             QSGPVS-T--FA-----GGQYSLKFTDV--SGTVRIDSLWTRTKVSSSVFS-----TDPV
FLA6             --NPVR-T--QATGQD-GGVFGLNFTGQAQSNQVNVSTGVVETRINNALRQ-----QFPL
FLA9             --NPVR-T--QASGRDN-GVYGLNFTGQ--TNQINVSTGYVETRISNSLRQ-----QRPL
FLA13            --NPVR-T--QASGRDVGGVYGLNFTGQ--GNQVNVSTGVVETRLSTSLRQ-----ERPL
FLA11            --NPLR-T--QAGDGQ-NGKFPLNITSS--GNQVNITTGVVSATVANSVYS-----DKQL
FLA12            --NPLR-T--QAGDSA-DGHFPLNVTTS--GNTVNITSGVTNTTVSGNVYS-----DGQL
FLA4Fas1-2       --NPVQPT--LATEEMGAGSYTLNISR-VNGSIVTINSGVVLAVVTQTAFD-----QNPV
FLA8Fas1-2       --NNIS-T--LATN--GAGKFDLTTS--TSGDEVILHTGVAPSRLADTVLD-----ATPV
FLA10Fas1-2      --DAIS-T--LATN--GAGKYDLTTS--TSGDEVILHTGVGPSRLADTVVD-----ETPV
FLA1Fas1-2       --GPMN-T--LATD--GANKFELTVQ--NDGEKVTLKTRINTVKIVDTLID-----EQPL
FLA2Fas1-2       --GAVN-T--LATE--GNNKFDFTVQ--NDGEDVTLETDVVTAKVMGTLKD-----QEPL
FLA4Fas1-1       PSGSAVTTLYEASGRTFFGSGSVNVTRDPASGSVTIGSPAT---KNVTVLKLLETKPPNI
FLA3             EKSTLLTTLYQSTGLGQQQNGFLNCTKS--NGKIYFGSGVKGAPQTAEYITTVFRNPYNL
FLA5             EKSIMLTTLYQTTGLGEQMNGFLNVSKS--KGRVYFGSEVKNSPLNAEYVSTVYHNPYNL
FLA14            KKTVLLTTLFQSSGLARGQQGFLNATVM-KNGDVAFGSAVPGSSLDAQLQDTVAALPFNI
FLA8Fas1-1       KGTTLSTTLYQTTGNAPGNLGFVNITDL-KGGKVGFGSAASGSKLDSSYTKSVKQIPYNI
FLA10Fas1-1      KGTTLTTTLYQTTGHALGNLGFVNVTDL-KGGKVGFGSAAPGSKLDSSYTKSVKQIPYNI
FLA1Fas1-1       DGSALAATLFQATGAAPGTSGFVNITDL-RGGKVGFGPDG--GDLSSFFVKSIEEVPYNI
FLA2Fas1-1       DGSTSTASMFQSTGSATGTSGYINITDI-KGGKVAFGVQDDDSKLTAHYVKSVFEKPYNI
                                                    .

                                        N135                  N154         N167
                                        N312  N317
```

supplemental Figure S9: Positioning of predicted N-glycosylation sites in Fas1 domains of *Arabidopsis thaliana* FLAs. The Fas1 domains of every *Arabidopsis thaliana* FLA were aligned using CLUSTAL and predicted N-glycosylation sites are boxed in purple. The residues with respect to the FLA4 sequence are indicated below the alignment. Note the low degree of sequence conservation outside the H1 and H2 regions (only the regions that contain predicted N-glycosylation sites are shown).
