# Supplementary material for: Arabidopsis thaliana FLA4 functions as a glycan‐stabilized soluble factor via its carboxy‐proximal Fasciclin 1 domain
Source: Plant J. 2017 Jun 13;91(4):613–30. doi: 10.1111/tpj.13591 (PMC5575511; doi:10.1111/tpj.13591)
Supplement: Supplementary file 11 — Figure S11. Detailed protein report on peptides identified in the two excised protein bands visible after F4C immuno‐affinity purification. [file TPJ-91-613-s011.pdf]

Detailed Protein Report

Project Info

**Name:**	20140128_Seifert	**Date:**	January 28, 2014

Sample Info & Protocols

		**Date:**	January 28, 2014
**Name:**	Gelbanden		

Search Result Info

Search Result	Location	Search Engine	Database	Ident. Compounds
IonTrap_allOrg_2014-01-31 14:28:43	/20140128_Seifert/Gelbanden/tryp_oben/ ProteinAnalysisResults.mgf	Mascot, 2.3.02	SwissProt, SwissProt_2011_12.fasta	108/7442

**Protein 1:**	Fasciclin-like arabinogalactan protein 4 OS=Arabidopsis thaliana GN=FLA4 PE=1 SV=1		
**Accession:**	FLA4_ARATH	**Score:**	384.83
**Database:**	SwissProt	**MW [kDa]:**	44.20
**Seq. Coverage [%]:**	13.80 %	**pI:**	5.50
		**No. of Peptides:**	13

10	20	30	40	50	60	70	80	90	100	110	120
MANVISISHF	TLLALPYLLL	LLSSTAAAIN	VTAVLSSFPN	LSSFSNLLVS	SGIAAELSGR	NSLTLLAVPN	SQFSSASLDL	TRRLPPSALA	DLLRFHVLLQ	FLSDSDLRRI	PPSGSAVTTL
**130**	**140**	**150**	**160**	**170**	**180**	**190**	**200**	**210**	**220**	**230**	**240**
YEASGRTFFG	SGSVNVTRDP	ASGSVTIGSP	ATKNVTVLKL	LETKPPNITV	LTVDSLIVPT	GIDITASETL	TPPPTSTSLS	PPPAGINLTQ	ILINGHNFNV	ALSLLVASGV	ITEFENDERG
**250**	**260**	**270**	**280**	**290**	**300**	**310**	**320**	**330**	**340**	**350**	**360**
AGITVFVPTD	SAFSDLPSNV	NLQSLPAEQK	AFVLKFHVLH	SYYTLGSLES	ITNPVQPTLA	TEEMGAGSYT	LNISRVNGSI	VTINSGVVLA	VVTQTAFDQN	PVSVFGVSKV	LLPKELFPKS
**370**	**380**	**390**	**400**	**410**	**420**	**430**					
GQPVATAPPQ	EISLSPESSS	EQPSRLVSPP	REIVSSGAVK	RPLGFLVLWC	WCIAFCYVLV						

Detailed Protein Report

Cmpd.	No. of Cmpds.	m/z meas.	Δ m/z [ppm]	z	Rt [min]	Score	P	Range	Sequence	Modification
3558	1	583.3755	42.66	2	28.4	25.7	0	84-94	R.LPPSALADLLR.F	
4065	2	845.4833	37.55	2	32.0	103.7	0	95-108	R.FHVLLQFLSDSDLR.R	
4072	2	845.4536	2.40	2	32.0	103.7	0	95-108	R.FHVLLQFLSDSDLR.R	
4111	5	564.3303	638.54	3	32.3	27.8	0	95-108	R.FHVLLQFLSDSDLR.R	
4043	5	564.0230	93.63	3	31.8	29.6	0	95-108	R.FHVLLQFLSDSDLR.R	
4133	5	563.9752	8.96	3	32.5	30.8	0	95-108	R.FHVLLQFLSDSDLR.R	
4122	5	564.0073	65.81	3	32.4	48.2	0	95-108	R.FHVLLQFLSDSDLR.R	
4102	5	564.0504	142.27	3	32.3	49.3	0	95-108	R.FHVLLQFLSDSDLR.R	
2293	2	621.4269	155.37	3	19.9	39.4	1	109-126	R.RIPPSGSAVTTLYEASGR.T	
2302	2	621.3877	92.17	3	20.0	45.8	1	109-126	R.RIPPSGSAVTTLYEASGR.T	
2540	4	853.4601	21.88	2	21.7	99.3	0	110-126	R.IPPSGSAVTTLYEASGR.T	
2048	2	694.2212	-195.32	2	18.4	36.0	0	139-153	R.DPASGSVTIGSPATK.N	
1954	2	694.3871	43.59	2	17.8	91.5	0	139-153	R.DPASGSVTIGSPATK.N	

supplemental Figure S11: Detailed protein report on peptides identified in the two excised protein bands visible after F4C immuno-affinity purification.
