# Supplementary material for: Arabidopsis thaliana FLA4 functions as a glycan‐stabilized soluble factor via its carboxy‐proximal Fasciclin 1 domain
Source: Plant J. 2017 Jun 13;91(4):613–30. doi: 10.1111/tpj.13591 (PMC5575511; doi:10.1111/tpj.13591)
Supplement: Supplementary file 14 — Table S1. Conserved N‐glycosylation sites at N‐terminal margin of Fas1 domains in Arabidopsis FLAs. [file TPJ-91-613-s014.docx]

**Supplemental Table S1**: Conserved N-glycosylation sites at N-terminal margin of Fas1 domains in Arabidopsis FLA peptides.

| **AtFLA #** |  | **GPI pred** | **# of Fas1 domains** | **group** | **N-glyco sites predicted^[[1]](#footnote-1)^** | **N-glyco site at margin of Fas1-1** | **positions of N-glyco sites rel. to 1st aa of H1 region (Fas1-1)** | **N-glyco site at margin of Fas1-2** | **position of N-glyco site rel. to 1st aa of H1 region (Fas1-2)** | **N-glyco site directly abutting H region^[[2]](#footnote-2)^** |
| --- | --- | --- | --- | --- | --- | --- | --- | --- | --- | --- |
| 6 | AT2G20520 | + | 1 | A | 5 | + | -37, -19 | na | na | - |
| 7 | AT2G04780 | + | 1 | A | 3 | + | -38 | na | na | - |
| 9 | AT1G03870 | + | 1 | A | 5 | + | -38, -19 | na | na | - |
| 11 | AT5G03170 | + | 1 | A | 4 | + | -39, -7 | na | na | - |
| 12 | AT5G60490 | + | 1 | A | 5 | + | -38, -6 | na | na | - |
| 13 | AT5G44130 | + | 1 | A | 5 | + | -38, -19, -6 | na | na | - |
| 3 | AT2G24450 | + | 1 | C | 3 | + | -33 | na | na | H2 |
| 5 | AT4G31370 | + | 1 | C | 4 | + | -33 | na | na | H2 |
| 14 | AT3G12660 | + | 1 | C | 4 | + | -33 | na | na | H2 |
| 19 | AT1G15190 | - | 1 | D | 2 | - | nf | na | na |  |
| 21 | AT5G06920 | - | 1 | D | 5 | + | -31,-1 | na | na | H1 |
| 15 | AT3G52370 | - | 2 | B | 2 | - | -1 | ?^[[3]](#footnote-3)^ | -18 | H1-1 |
| 16 | AT2G35860 | - | 2 | B | 2 | - | -1 | ? | -18 | H1-1 |
| 17 | AT5G06390 | - | 2 | B | 2 | - | -1 | ? | -18 | H1-1 |
| 18 | AT3G11700 | - | 2 | B | 4 | ? | -55,-45,-1 | ? | -18 | H1-1 |
| 1 | AT5G55730 | + | 2 | C | 5 | + | -33 | + | -35 | H2-1 |
| 2 | AT4G12730 | + | 2 | C | 4 | + | -33 | - | nf | H2-1 |
| 8 | AT2G45470 | + | 2 | C | 5 | + | -33 | + | -34 | H2-1 |
| 10 | AT3G60900 | + | 2 | C | 5 | + | -33 | + | -34 | H2-1 |
| **4** | **AT3G46550** | **+** | **2** | **D** | **8** | **+** | **-33, -23** | **+** | **-36** | **H2-1** |
| 20^[[4]](#footnote-4)^ | AT5G40940 | - | 2 | D | 6 | ? | -50 | ? | -45,-8,-4 |  |

1. all NXT/S [↑](#footnote-ref-1)
2. H1-2 indicates the H1 domain of Fas1-2 etc. [↑](#footnote-ref-2)
3. In cases noted by "?" the position of an N-terminal N-glyco site is too diverged from the consensus for confident annotation [↑](#footnote-ref-3)
4. The annotation of AtFLA20 as FLA is problematic due to the obvious lack of an N-terminal secretion signal and GPI modification motif. [↑](#footnote-ref-4)
