# Supplementary material for: Arabidopsis thaliana FLA4 functions as a glycan‐stabilized soluble factor via its carboxy‐proximal Fasciclin 1 domain
Source: Plant J. 2017 Jun 13;91(4):613–30. doi: 10.1111/tpj.13591 (PMC5575511; doi:10.1111/tpj.13591)
Supplement: Supplementary file 15 — Table S2. Oligonucleotide primers used in this study. [file TPJ-91-613-s015.docx]

Supplemental Table 2: Oligonucleotide primers used in this study

| FLA4Pro-SacII-F | ataCCGCGGAAATAAGTACACACTAACCTTG | all pFLA4 constructs |
| --- | --- | --- |
| FLA4Pro-SphI-R | ataGCATGCTATTTTTTGAGTTAGGTCATGAG | all pFLA4 constructs |
| UBQ10p-SacII-F | aaCCGCGGgtcgacgagtcagtaataaacg | all pUBQ10 constructs |
| UBQ10pSphI-R | aaGCATGCtgttaatcagaaaaactcagatta | all pUBQ10 constructs |
| RSP5A-SacII-F | aaCCGCGGtacatcaaggatatgtccttctgaa | all pRSP5 constructs |
| RsP5A-SphI-R | aaGCATGCaatggctgtggtgagagaaacagag |  |
| Sph1-FLA4-NSP-F | acatGCATGCAGATGGCGAACGTAATCTCAATT | all constructs |
| Xma1-FLA4-NSP-R | cccCCCGGGTGCGGCGGCGGTGGAAGA | all constructs |
| citrin-Xma-F | CCCCGGGTAATGGTGAGCAAGGGCGAG | all constructs |
| citrin-BsrGI-R | ACTTGTACAGCTCGTCCATG | all constructs |
| BsrGI-FLA4-F | ACATTGTACAAGATTAACGTCACCGCCGTCCTC | all F4C constructs |
| Not1-FLA4-R | ataagaatGCGGCCGCTCATACCAAAACATAACAAAATG | all F4C constructs |
| HSP18.2t/NotI-F | aaGCGGCCGCATATGAAGATGAAGATGAAATATTTG | all constructs |
| HSP18.2t/ApaI-R | aaGGGCCCATCTTATCTTTAATCATATTCCATAG | all constructs |
| BsrG1-PR1-F | ttacatTGTACAAGGACTCCCTCATCGTCCCCA | pFLA4::F4C∆Fas1-1 |
| BsrGI-FLA4delPR1.1-F | atTGTACAAGGGAATCGATATCACCGCA | pUBQ10::F4C∆Fas1-1∆PR1.1 |
| BsrGI-FLA4delPR1.2-F | atTGTACAAGGAGACTCTCACTCCACC | pUBQ10::F4C∆Fas1-1∆PR1.1 |
| BsrGI-FLA4delPR1.3-F | atTGTACAAGACGTCAACATCTCTCTCC | pUBQ10::F4C∆Fas1-1∆PR1.1 |
| BsrGI-FLA4delPR1.4-F | atTGTACAAGGCGGGAATCAATCTCAC | pUBQ10::F4C∆Fas1-1∆PR1.4 |
| BsrGI-FLA4delPR1.5-F | atTGTACAAGCTCACTCAGATACTAATCAAC | pUBQ10::F4C∆Fas1-1∆PR1.2 |
| BsrG1-FL2-F | ttacatTGTACAAGGGACACAACTTCAACGTCGCT | pFLA4::F4C∆Fas1-1∆PR1 |
| BsrG1-PR2-F | ttacatTGTACAAGCCTAAAGAACTATTTCCAAAAT | pFLA4::F4C∆Fas1-1∆PR1∆Fas1-2 |
| FLA4-N207Q-F | AATCCAACTCACTCAGATACTAATCAACGGAC | pFLA4::F4CN207Q |
| FLA4-N207Q-R | TGAGTGAGTTGGATTCCCGCC | pFLA4::F4CN207Q |
