# Supplementary material for: Arabidopsis thaliana FLA4 functions as a glycan‐stabilized soluble factor via its carboxy‐proximal Fasciclin 1 domain
Source: Plant J. 2017 Jun 13;91(4):613–30. doi: 10.1111/tpj.13591 (PMC5575511; doi:10.1111/tpj.13591)
Supplement: Supplementary file 16 [file TPJ-91-613-s016.docx]

**Supporting Information Legends**

**Figure S1.** FLA4-citrin sequence, domains and key features. N-terminal signal peptide predicted by SignalP4.1 (underlined). mCitrin (yellow), N-proximal Fas1-1 domain (dark green), C-proximal Fas1-2 domain (light green), H-regions (pink), N-glycosylation sites (NXS/T; purple), Ser mutated to Phe in sos5-1 (red) clustered Pro-residues in Pro-rich regions (olive), GPI-modification signal (weakly) predicted by BIG-PI Plant predictor (grey) are highlighted.

**Figure S2**. Treatment with GPI-PLC removes F4C and SKU5 from the membrane fraction. Two phase partitioning of microsomes was followed either by mock incubation at 29°C for 90 minutes (-) or in the presence of GPI-PLC (+). Mock treatment alone showed predominant presence of F4C in the aqueous phase (A) compared to the detergent phase (M). When GPI-PLC was present the overall signal was strongly reduced and F4C was only detectable in the soluble phase. SKU5 detected in the same fractions showed approximately equal abundance in soluble and detergent phase after mock treatment and was only detectable in the soluble phase after enzyme treatment. The presence of previously membrane anchored proteins in the aqueous phase after the mock treatment suggests presence of endogenous lipases. Why GPI-PLC strongly reduced the signal for the fluorescent protein tag is unknown.

**Figure S3**. The C-terminal putative GPI-modification signal sequence is not required for genetic function of F4C. To show complementation of the sos5-1 mutant, three independent homozygous lines transformed with either full length FLA4p:F4C (yellow) or C-terminally truncated FLA4p:F4C∆GPI (orange) were compared with respect to root length and the root thickness at the level of root hair differentiation on NaCl free control medium (MS0) 6 days after germination (dpg), or root growth or thickness after 2 days on medium supplemented with 100mM NaCl. To test whether the transgene might behave differently in the T-DNA insertion mutant sos5-2, one complementing line (in the sos5-1 background) was crossed with the sos5-2 mutant and F3 populations homozygous for the sos5-2 allele and for the transgene were tested. Error bars indicate the ±5% confidence intervals.

**Figure S4**. Alignment of putative FLA4 orthologues. The highly conserved H1 and H2 regions are underlined in the FLA4 sequence. Predicted N-glycosylation sites are boxed purple. Proline residue in PR regions are boxed olive.

**Figure S5**. Peptide sequence polymorphisms in AtFLA4 in various Arabidopsis accessions revealed by the 1001 genomes project. The Col-0 sequence is boxed green in the two Fas1 domains and pink in the H1 and H2 domains. Predicted N-glycosylation sites are boxed purple. Clustered proline residure in PR regions are boxed olive. Polymorphic residues are boxed yellow with the substituted residues shown underneath. The table below summarizes the occurrence of non-synonymous polymorphisms in the various functional domains of AtFLA4 by degree of conservation according to CLUSTAL. Note the relatively high degree of conservation in the entire Fas1-2 domain.

**Figure S6**. A) N-proximal Fas1-1 region and the PR1 domain are not required for complementing sos5-1. For comparison, the consensus among putative angiosperm FLA4 orthologues and of the closest Arabidopsis FLA4 paralogue AtFLA8 are shown. B) For assessing complementation of genetic FLA4 function in unstressed roots, root length after 5 days on MS0 medium is shown in duplicate homozygous lines. C) To assess complementation of NaCl tolerance root elongation after 2 days on 100mM NaCl is shown.

**Figure S7**. Brefeldin A leads to redistribution of F4C and F4C∆Fas1-1 into BFA bodies, while F4C∆Fas1-1∆PR1.6 is not affected. 25µM BFA in H2O was applied for 50 minutes.

**Figure S8**. Localization of several constructs that lack the Fas1-1 domain and to different extent the PR1 domain. The F4C wild type is mostly membrane localized. The F4C∆Fas1-1 and F4C∆Fas1-1∆PR1.4 that both lack the Fas1-1 domain and retain or lack the PR1 domain, respectively show similar localization in endosomal structures, and cell corners as well as the cell surface. The F4C∆Fas1-1∆PR1.5 and F4C∆Fas1-1∆PR1.6 constructs are both mainly localized to ER-like structures.

**Figure S9**. Positioning of predicted N-glycosylation sites in Fas1 domains of Arabidopsis thaliana FLAs. The Fas1 domains of every Arabidopsis thaliana FLA were aligned using CLUSTAL and predicted N-glycosylation sites are boxed in purple. The residues with respect to the FLA4 sequence are indicated below the alignment. Note the low degree of sequence conservation outside the H1 and H2 regions.

**Figure S10**. The N-glycosylation site N207 is not required for F4C function in root growth and NaCl tolerance. Two independent homozygous transformant lines in the sos5-1 background and line #1 outcrossed to sos5-2, homozygous for sos5-2 and for the transgene, complement normal root growth and root thickness on MS0 (5dpg) and on 100mM NaCl (2dpi). Error bars indicate ±5% confidence intervals. Note that the outcrossed line homozygous for sos5-2 showed reduced growth on MS0. As this was accompanied with wild type-like root thickness on MS0 and wild-type like grow and thickness on NaCl this construct complements FLA4 function in the sos5-2 background. The reduced root growth on MS0 could be due to interactions between Col gl and Col-0 wild type backgrounds.

**Figure S11**. Detailed protein report on peptides identified in the two excised protein bands visible after F4C immuno-affinity purification.

**Figure S12.** Inhibitor of prolyl4-hydroxylase bipyridyl (BP) suppresses F4C abundance. BP after 5 hr exposure of seedlings in liquid culture of FLA4:F4C and untransformed controls (Col-0). Ponceau S stain is shown as loading control.

**Figure S13**. The clustered proline residues in the two PR domains are not required for FLA4 function in root growth and NaCl tolerance. Three independent transformant lines in the sos5-1 background and outcrossed line #5 in the homozygous sos5-2 background show wild-type like growth behaviour with respect to root growth and thickness on NaCl-free MS0 and MS0 containing 100mM NaCl.

**Table S1.** Conserved N-glycosylation sites at N-terminal margin of Fas1 domains in Arabidopsis FLAs.

**Table S2.** Oligonucleotide primers used in this study
